# From gut-reproductive microbiota to ferroptosis: a comprehensive insight into the molecular-pathogenicity of endometriosis

**DOI:** 10.3389/fimmu.2026.1762013

**Published:** 2026-05-22

**Authors:** Xianhua Han, Xin lei Guo, Junyu Qiu

**Affiliations:** 1 Department of Gynecology, Ganzhou Hospital-Nanfang Hospital, Southern Medical University (Ganzhou People’s Hospital), Ganzhou, Jiangxi, China; 2 Gannan Medical University, Ganzhou, Jiangxi, China; 3 Department of Ultrasound, The First Affiliated Hospital of Gannan Medical University, Ganzhou, Jiangxi, China

**Keywords:** endometriosis, ferroptosis, gut microbiome, immune microenvironment, reproductive microbiome, therapeutic strategies

## Abstract

Endometriosis (EMS) is a highly heterogeneous chronic gynecological disease characterized by pain, infertility, and relapse, with its etiology and pathogenesis not yet fully elucidated. Traditional theories, including “retrograde menstruation,” “implantation theory,” and “abnormalities in immune tolerance,” struggle to adequately explain the complex lesion behavior, diverse phenotypic characteristics, and accompanying immune-metabolic disorders. In recent years, the key roles of imbalances in the gut and reproductive microbiomes, abnormal iron metabolism, and the newly proposed ferroptosis in the occurrence and development of EMS have gradually gained attention, suggesting that this disease may be a systemic condition involving the interplay of microbial ecology, iron metabolism, and cell death. Existing studies indicate that the gut-reproductive microbiome profoundly influences the body’s iron homeostasis and iron load by regulating mucosal immunity, systemic inflammatory responses, and metabolic environments. This, in turn, activates the ferroptosis pathway through iron-dependent lipid peroxidation and cell membrane damage, participating in the formation, maintenance, and inflammatory microenvironment shaping of ectopic lesions. Based on these findings, this article systematically reviews the interactions between gut-reproductive microbiome imbalance and iron metabolism disorders, integrating multi-omics evidence such as microbiome analysis, metabolomics, and iron metabolism/ferroptosis-related molecular markers. It proposes a new pathological mechanism framework of “dysbiosis–iron overload–ferroptosis” incorporating microecological imbalance and ferroptosis into a unified picture of the pathogenesis of EMS. Furthermore, this article discusses potential therapeutic strategies and application prospects surrounding microbiome remodeling (such as probiotics, fecal microbiota transplantation, dietary and lifestyle interventions) and pharmacological targeting of key ferroptosis-related molecules. Through a comprehensive and critical analysis of existing evidence, this review aims to provide a more systematic theoretical framework for the mechanistic research of EMS and offer ideas and directions for future clinical translation of precise classification, individualized intervention, and novel treatment plans.

## Graphical Abstract

**Figure:**
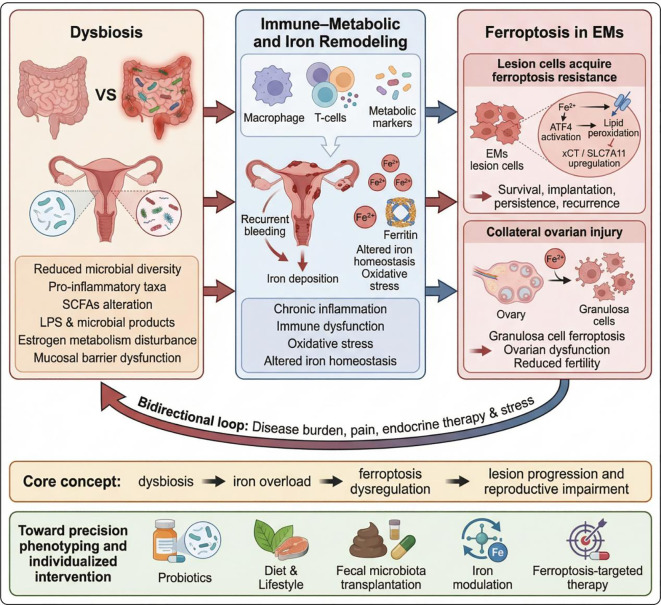
The microbiota–iron metabolism–ferroptosis axis in endometriosis. This image summarizes the central conclusion of the review: in EMS, gut and reproductive tract dysbiosis may promote immune–metabolic remodeling and iron homeostasis disturbance through barrier dysfunction, inflammatory microbial products, altered SCFAs, and disordered estrogen metabolism. Recurrent bleeding and iron deposition further amplify oxidative stress, chronic inflammation, and immune dysfunction. Within ectopic lesions, endometriotic cells may acquire ferroptosis resistance, thereby supporting lesion survival, implantation, persistence, and recurrence; in parallel, ferroptosis-related injury may also affect ovarian granulosa cells, contributing to ovarian dysfunction and reduced fertility. Overall, EMS can be conceptualized as a bidirectional pathogenic loop driven by dysbiosis, iron overload, and ferroptosis dysregulation, providing a rationale for precision phenotyping and individualized interventions based on microbiota modulation, iron regulation, and ferroptosis-targeted therapy.

## 1 Introduction

Endometriosis (EMS) is a common chronic gynecological disease, primarily characterized by the ectopic growth of tissue resembling endometrium outside the uterine cavity, such as on the ovaries and peritoneum. This leads to clinical symptoms including pain, menstrual abnormalities, and infertility; these symptoms severely affect the quality of life of women (1, 2). It is estimated that approximately 5 to 10 percent of women of childbearing age worldwide are affected by this disease. The clinical manifestations and disease progression show high heterogeneity in symptoms, lesion types, and progression rates (3). Traditional pathophysiological theories, such as the theory of retrograde menstruation and the implantation theory, provide basic explanations for the occurrence of EMS but do not fully explain the complex clinical heterogeneity and mechanisms underlying systemic immune dysfunction (4).

In recent years, with advances in molecular biology and multi-omics technologies, researchers have increasingly focused on changes in the microenvironment of EMS, abnormalities in immune regulation, and various forms of cell death. These factors all contribute to disease development. Among the pathological mechanisms of EMS, imbalance in the gut and reproductive tract microbiomes is increasingly recognized as a key factor. The gut and reproductive tract, as important microbial ecosystems of the body, undergo changes in their microbial community structures. These changes not only affect local immune status but also regulate the host’s inflammatory response and hormone metabolism through microbial metabolic products, thereby influencing the formation and maintenance of EMS lesions (5). Studies have shown that the diversity of gut and reproductive tract microbiota in EMS patients is significantly reduced, accompanied by enrichment of inflammation-related microbiota. This microbial imbalance may promote an inflammatory microenvironment in the lesions, further exacerbating disease symptoms (6, 7). Additionally, gut microbes can influence iron metabolism and the redox state of host cells through their metabolic products, such as short-chain fatty acids (e.g.,butyrate), thereby regulating cell death modalities, particularly iron-dependent programmed cell death (ferroptosis). Butyrate has been found to increase the susceptibility of EMS cells to ferroptosis, suggesting that microbial metabolites play a regulatory role in the pathogenesis of EMS (8).

Ferroptosis is a novel form of programmed cell death characterized by iron-dependent lipid peroxidation reactions, which lead to the destruction of the lipid bilayer of cell membranes and ultimately result in cell death (9, 10). Ferroptosis is involved in various pathological processes, including cancer and neurodegenerative diseases, and also plays significant roles in gynecological diseases such as EMS (11). Lesions of EMS are often accompanied by local iron overload. This overload results from repeated bleeding within the lesions, which causes iron deposition and subsequently activates ferroptosis-related pathways. In contrast, EMS cells exhibit a certain resistance to ferroptosis. This resistance may be due to the activation of specific transcription factors such as Activating Transcription Factor 4 (ATF4), which enhances the expression of antioxidant systems, for example, the cystine/glutamate antiporter xCT, thereby helping lesion cells resist ferroptosis and maintain their survival and continued development (12, 13). Moreover, ferroptosis induced by iron overload not only affects EMS lesion cells but also damages ovarian granulosa cells, leading to decreased ovarian function and impaired fertility (14, 15). Therefore, ferroptosis plays a dual role in the pathological process of EMS: it participates in the formation and maintenance of lesions while simultaneously affecting patients’ reproductive function. Currently, research on the association between gut-reproductive tract microbial imbalance, iron metabolism disorders, and ferroptosis in EMS remains fragmented and lacks systematic integrated reviews. Most studies have approached this issue from the perspective of microbial community changes or ferroptosis mechanisms, failing to form a comprehensive map of the overall pathological axis from gut microecology and iron metabolism to ferroptosis (16, 17).

This article aims to synthesize microbiome dysbiosis, abnormal iron metabolism, and ferroptosis mechanisms within a unified framework to explore their intersecting roles and potential clinical significance in the pathological progression of EMS. Notably, although existing evidence supports a close association between EMS and abnormalities in the gut–reproductive tract microbiome, the question of whether “microbial dysbiosis is an upstream driver of EMS or a secondary phenomenon resulting from local inflammation, hormonal environmental changes, and recurrent bleeding caused by the disease” has not been fully elucidated. Based on current human correlation studies, animal experiments, and mechanistic inferences, current evidence suggests that these processes do not exhibit a unidirectional causal relationship but may form a bidirectional, mutually reinforcing pathological loop through immune imbalance, metabolic reprogramming, and iron homeostasis abnormalities. Consequently, this study expands upon these findings by integrating the conceptual framework of “dysbiosis–iron overload–ferroptosis” to elucidate the complex pathological mechanisms of EMS from a more holistic perspective and to provide a theoretical basis for future precise classification and individualized intervention.

## 2 Imbalance of gut and reproductive microbiome in EMS

### 2.1 Changes in gut microbiome

Patients with EMS may exhibit significant alterations in their gut microbiome, primarily characterized by reduced microbial diversity and altered relative abundances of certain bacterial groups. Studies have shown differences in the gut microbiota structure between EMS patients and healthy controls, particularly in the relative abundances of common bacterial genera such as Bacteroides and Prevotella (18, 19). Some studies have also observed a decrease in the abundance of Firmicutes and an increase in Proteobacteria within the gut microbiota of patients with EMS, indicative of dysbiosis (20). Additionally, certain opportunistic pathogens, such as Escherichia coli and Klebsiella pneumoniae, have been found to be relatively enriched in some patients (21, 22).

Beyond the overall decline in diversity, changes in the gut microbiota associated with EMs can also manifest as a trend of “decreased beneficial commensal bacteria and increased opportunistic pathogens.” For example, genera such as faecalibacterium, which are associated with healthy gut homeostasis, have been reported to be depleted in some studies and show a negative correlation with disease severity (23, 24). Notably, differences in sample sources, patient subtypes, disease stages, diet, and prior treatments across studies account for heterogeneity in specific microbiota changes among different cohorts. Overall, the more consistent observation is that EMS patients exhibit detectable compositional shifts in their gut microbiome, with key features including reduced microbial diversity and altered relative abundances of certain bacterial groups.

In summary, abnormalities in the gut microbiome of EMS patients are primarily reflected in changes in microbial composition and relative abundances, providing foundational evidence for understanding microbiota-related dysbiosis in EMS. The potential immunological and metabolic implications of these changes will be further discussed in Section 3.

### 2.2 Vaginal and reproductive tract microbiome

The vaginal and genital tract microbiome plays a significant role in female reproductive health, characterized by the dominance of Lactobacillus species, notably including L. crispatus, L. gasseri, and L. jensenii (25, 26). In a healthy state, this Lactobacillus-dominated community structure is typically associated with lower vaginal pH and a more stable local environment (27).

When the vaginal microbiome becomes imbalanced, it often manifests as a reduction in Lactobacillus and an increase in non-Lactobacillus or anaerobic bacteria such as Gardnerella vaginalis, Atopobium vaginae, and Prevotella spp (28, 29). Studies also indicate that increased vaginal microbial diversity is often accompanied by a decrease in Lactobacillus and is frequently associated with elevated vaginal pH (30). In patients with EMS, such shifts in the genital tract microecology are more common. Multiple studies suggest that in vaginal and related genital tract samples from EMS patients, the abundance of Lactobacillus decreases, while bacteria such as Gardnerella, Streptococcus, Escherichia coli, and Mycoplasma increase (31–33). Additionally, some studies have observed increased vaginal microbial diversity in EMS patients, with a tendency to shift from a “Lactobacillus-dominated” state to “polymicrobial dysbiosis” (34, 35). Differences in sampling sites, detection platforms, and study populations across various studies lead to inconsistencies in findings regarding specific dominant or pathogenic bacteria. However, overall, the changes in the genital tract microbiome associated with EMS show a consistent directional trend, namely a reduction in Lactobacillus and relative enrichment of opportunistic pathogens.

In summary, the imbalance in the vaginal and genital tract microbiome is a key component of microecological abnormalities related to EMS, primarily characterized by a weakened dominance of Lactobacillus and an increase in various non-Lactobacillus species. The specific immune and metabolic mechanisms will be discussed in Section 3.

### 2.3 The association between the microbiome and EMS pain and female infertility

**Figure 1:**
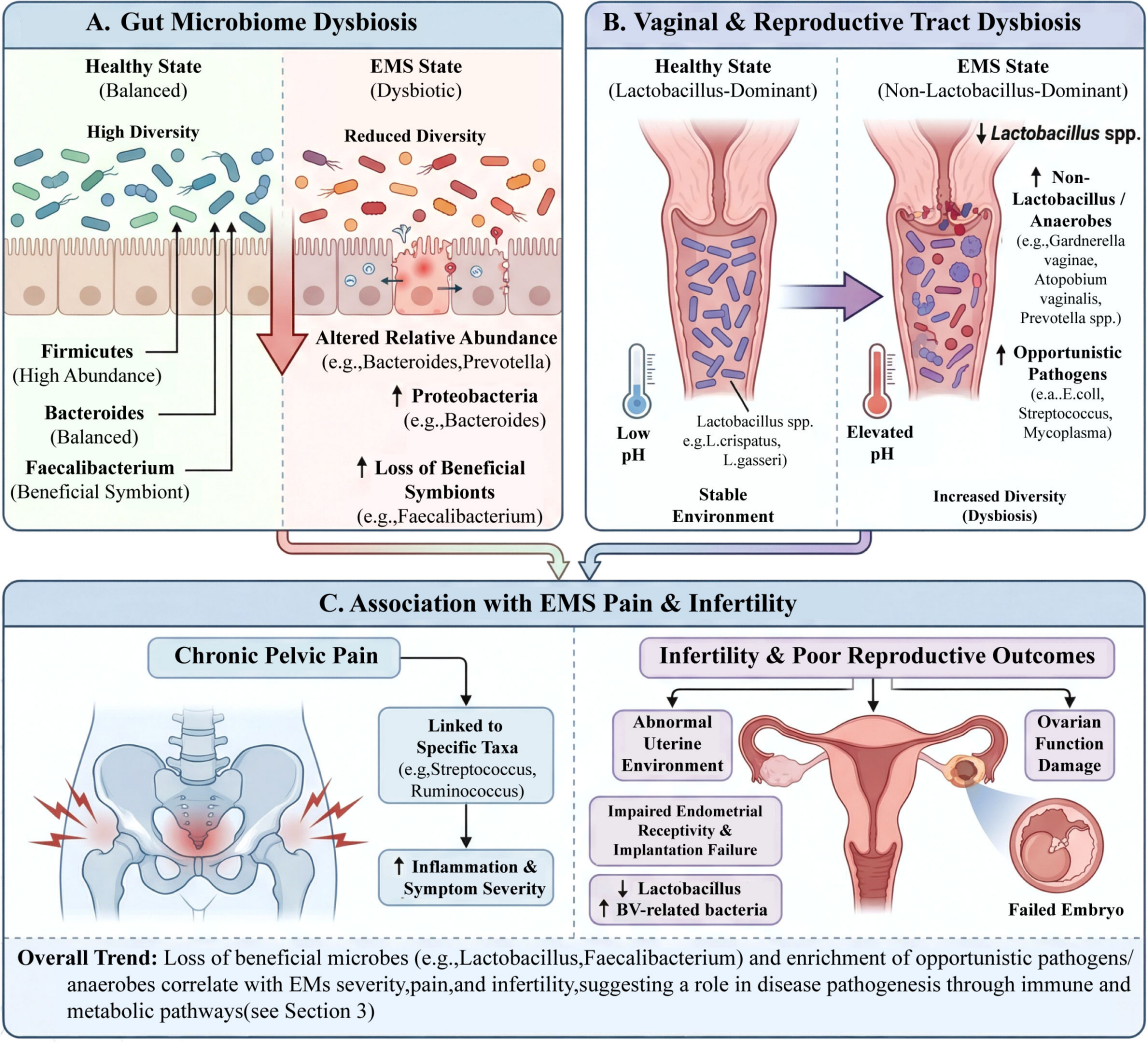
Microbiota imbalance in EMS: the gut–reproductive tract axis and its clinical associations. **(A)** At the gut level, this manifests as reduced microbial diversity, decreased beneficial commensal bacteria, and a relative increase in opportunistic pathogens **(B)** at the vaginal and reproductive tract levels, it presents as reduced Lactobacillus abundance, an increase in anaerobic and opportunistic pathogens, accompanied by elevated local pH and disruption of microenvironment homeostasis. **(C)** This microecological disturbance across the gut–reproductive tract axis is associated with chronic pelvic pain, increased inflammation, infertility, and adverse reproductive outcomes, suggesting that microbiota imbalance may contribute to the pathogenesis and progression of EMS through immune and metabolic pathways. (EMS:Endometriosis). This image was drawn using BioRender software (https://app.biorender.com).

The main clinical manifestations of EMS include pain and infertility, and an increasing number of studies suggest an emerging link between microbiome changes and these phenotypes (36). In pain-related research, patients with chronic pelvic pain often exhibit decreased diversity in vaginal or gut microbiota and abnormal abundance of certain microbial populations (34). For example, bacteria such as Streptococcus anginosus and Ruminococcus exhibit increased abundance in some pain-related cohorts and are associated with higher inflammatory markers or more severe symptoms (37, 38). Regarding reproductive outcomes, alterations in the vaginal, cervical, and gut microbiomes are also linked to infertility phenotypes. Studies indicate that a reduction in Lactobacillus species and an increase in bacteria associated with bacterial vaginosis are correlated with abnormal intrauterine environments and adverse reproductive outcomes in EMS patients (39–42). Additionally, some studies have found that EMS patients with higher cervical microbial diversity may exhibit lower pain scores or relatively better clinical outcomes, while microbiome dysbiosis may be associated with impaired ovarian function, decreased endometrial receptivity, and embryo implantation failure (43, 44). It is important to emphasize that most current evidence primarily comes from correlational studies, suggesting an association between microbiota alterations and pain or infertility phenotypes, but it is insufficient alone to support definitive causal conclusions. Overall, existing research supports a clinical correlation between EMS-related pain and infertility and specific microbiome characteristics, providing foundational clues for subsequent mechanistic studies and patient stratification (**Figure 1**).

## 3 The impact of gut-reproductive tract microbiota on the immune and metabolic axis

### 3.1 Microbiome and the intraperitoneal immune microenvironment

As mentioned in Section 2, EMS-related microecological abnormalities involve not only the gut but also the reproductive tract. Based on these compositional changes, an increasing number of studies are focusing on their impact on the intraperitoneal immune microenvironment. Gut microbiota can interact with host immune cells, such as macrophages and T cells, through metabolites and bacterial components, regulating their function and distribution, thereby shaping a specific immune microenvironment (45, 46). In EMS patients, immune cells in the peritoneal fluid exhibit significant alterations, such as a reduction in T cell subsets—specifically CD3^+^, CD4^+^, and CD8^+^ T cells—as well as natural killer (NK) cells, indicating the presence of an immunosuppressive state (47, 48). This may reflect how gut microbiota, by modulating the activity of immune cells, affects intraperitoneal immune monitoring and disease resistance.

In addition to gut microbiota, reproductive tract microecological imbalance may also contribute to the remodeling of the intraperitoneal immune microenvironment. Studies have observed a trend of reduced Lactobacillus and increased opportunistic or anaerobic pathogens such as Gardnerella, Atopobium, Streptococcus, Escherichia/Shigella, Prevotella, and Pseudomonas in cervical/vaginal secretions, endometrial samples, or peritoneal fluid of EMS patients (49). These microbial changes are not universally consistent across all studies but overall suggest a shift from “Lactobacillus dominance” to “polymicrobial dysbiosis” in EMs. Such changes can activate the TLR2/TLR4-NF-κB pathway through bacterial lipopolysaccharide (LPS), peptidoglycan, and other pathogen-associated molecular patterns (PAMPs), induce NLRP3 inflammasome activation, and promote the release of pro-inflammatory mediators such as IL-1β, IL-6, and TNF-α, thereby amplifying chronic intraperitoneal inflammation and impairing immune clearance (50).

Furthermore, gut microbiota imbalance (dysbiosis) is common in EMS, and alterations in the microbial community structure of the gut and female reproductive tract may lead to functional abnormalities in immune cells, promoting the formation of a local chronic inflammatory environment (51). Microbiota-induced chronic inflammation is one of the key factors sustaining endometriotic lesions. Elevated levels of inflammatory mediators such as cytokines, chemokines, and reactive oxygen species (ROS) in the peritoneal fluid reflect a persistent inflammatory state. Studies have found that specific microbes, such as Flavobacterium, Pseudomonas, and Bacillus, are enriched in the peritoneal fluid of EMS patients. These strains may activate Toll-like receptor (TLR) signaling pathways, induce inflammasome activation, and promote the secretion of pro-inflammatory cytokines, thereby exacerbating local inflammatory responses (52). This chronic inflammation not only supports the survival and proliferation of ectopic endometrial cells but can also lead to immune escape by disrupting immune tolerance mechanisms, further promoting lesion expansion and disease progression.

Notably, aryl hydrocarbon receptor (AhR) may not function solely as an exogenous chemical sensor in this intraperitoneal immune microenvironment (53). As a ligand-dependent transcription factor, AhR can integrate signals from environmental ligands, host endogenous metabolites, and some microbially derived molecules, and interact with inflammatory and oxidative stress pathways (54). Studies have observed increased AhR-positive mast cells in EMS lesion tissues, accompanied by active 2,3-dioxygenase 1(IDO1)–kynurenine pathway, IL-17 production, and increased ROS, suggesting that AhR may be involved in sustaining and amplifying local chronic inflammation in lesions (55). More importantly, AhR antagonism can alter the cytokine environment in lesion tissues and affect stromal cell growth, indicating that it is not merely a bystander molecule but may serve as a key regulatory node connecting microbial metabolism, immune cell activation, and sustained inflammation (56). Therefore, EMS-related pathogenic microecology should be understood as a “gut–reproductive tract” dual-source network, rather than being driven solely by gut microbiota: gut microbiota exerts remote effects primarily through systemic immune regulation, metabolite output, and estrogen recycling, while reproductive tract and local intraperitoneal microbiota more directly participate in mucosal barrier disruption, local inflammation amplification, immune escape, and lesion adhesion/invasion processes (57, 58). Additionally, crosstalk between the gut and reproductive tract microbiomes is believed to play a critical role in immune regulation in EMS. Gut microbiota influences systemic immune status and local immune environments, modulating intraperitoneal immune cell responses and inflammation levels (59). Environmental factors such as endocrine disruptors can indirectly affect disease progression by altering microbial communities and immune status (60).

In summary, gut and reproductive tract microbiota do not function in isolation but collectively shape the intraperitoneal immune microenvironment in EMS: the former influences systemic and distal immunity through metabolites, endotoxins, and estrogen recycling, while the latter directly promotes chronic inflammation and immune escape through local microbiota imbalance, PAMP stimulation, and barrier disruption. These two systems are intricately coupled, constituting an important microecological basis for the pathological progression of EMS.

### 3.2 Microbial metabolites

In recent years, the role of gut microbiota and their metabolites in regulating local host immune responses and cellular metabolism has garnered widespread attention, particularly in the pathogenesis of gynecological diseases such as EMS, in which they exert a significant influence (61). Gut microbiota produce a variety of active metabolites through the metabolism of dietary components and host secretions, with short-chain fatty acids (SCFAs), bile acids (BAs), and indole metabolites being the primary focus of research. These metabolites not only maintain intestinal homeostasis but also regulate the reproductive tract microenvironment and immune responses at distant sites (62, 63).

In addition to the aforementioned gut-derived metabolites, the dominant microbiota in the reproductive tract can also form an important local metabolic background. For example, lactic acid produced by Lactobacillus species not only helps maintain the acidic environment of the vagina, limiting the adhesion and ascending colonization of opportunistic pathogens, but also modulates the inflammatory phenotypes of epithelial cells, dendritic cells, and macrophages, thereby supporting local mucosal immune homeostasis. Conversely, when Lactobacillus decreases and anaerobic bacteria proliferate, the local acidic barrier weakens, creating a metabolic environment more conducive to inflammation, which provides favorable conditions for the chronic inflammation associated with EMs.

First, SCFAs, primarily including acetate, propionate, and butyrate, are key products of dietary fiber fermentation by gut anaerobic bacteria (64). SCFAs regulate the differentiation and function of immune cells by binding to their specific receptors, such as G protein-coupled receptors (GPR41, GPR43), and inhibiting histone deacetylase (HDAC) activity. This promotes the generation of regulatory T cells (Tregs) and suppresses the release of inflammatory cytokines, thereby creating an anti-inflammatory environment locally (65). In EMS, SCFAs have been found to exhibit anti-proliferative and anti-inflammatory effects, inhibiting the survival of ectopic endometrial cells and the growth of lesions, suggesting their protective role in EMS by modulating the immune microenvironment (66, 67). Additionally, SCFAs can further regulate local tissue homeostasis by influencing host energy metabolism and apoptosis pathways (68). Second, BAs are steroid metabolites synthesized by the liver and modified by gut microbiota to form secondary bile acids. These metabolites act as signaling molecules involved in regulating metabolism and immune function (69). BAs regulate intestinal barrier function and immune responses by activating nuclear receptors (e.g., farnesoid X receptor (FXR), TGR5), promoting the activation of anti-inflammatory cells and suppressing pro-inflammatory responses (70, 71). In the context of EMS, dysregulation of bile acid metabolism may contribute to hormonal imbalances and immune abnormalities, thereby promoting disease progression (72). Furthermore, bile acid metabolites can influence the composition of the gut microbiota, forming a feedback regulatory loop (73). Third, indole metabolites, such as indole-3-lactic acid, indole-3-propionic acid, and indole-3-aldehyde, are primarily derived from the microbial metabolism of tryptophan. These metabolites regulate intestinal mucosal immune homeostasis by activating the AhR and other signaling pathways, promoting the differentiation of anti-inflammatory cells and maintaining barrier function (74, 75). Indole metabolites also play a significant role in endometriosis, not only regulating local immune responses but also potentially influencing estrogen metabolism and inflammatory processes (76, 77).

It is important to further emphasize that AhR should not be narrowly understood as a “receptor for microbial indole metabolites” or merely as a xenobiotic sensor. More accurately, AhR is a ligand-dependent transcription factor capable of integrating signals from microbial-derived metabolites, host tryptophan metabolites, and environmental ligands, and interacting with pathways related to inflammation, oxidative stress, and tissue remodeling (78). In EMS, the enhancement of the IDO1–kynurenine axis offers compelling endogenous evidence of AhR activation within lesions. Concurrently, AhR activation may not only modulate the Treg/Th17 balance and local mucosal immunity but also contribute to the maintenance of chronic inflammatory states in lesions by promoting the production of IL-17, ROS, and other inflammatory mediators (79, 80). Recent animal studies also suggest that AhR-related interventions, in addition to affecting inflammatory responses, may be accompanied by changes in TGF-β-related signaling and oxidative stress states, indicating that the role of AhR in EMS may extend beyond the classical “microbial metabolite–immune regulation” framework to include fibrotic tendencies and antioxidant defense networks (81). However, this aspect in EMS is still primarily supported by animal studies and mechanistic inferences, lacking sufficient human functional validation. Therefore, from the perspective of EMS pathogenesis, SCFAs, bile acids, lactic acid, and indole metabolites are not isolated metabolic events but collectively constitute the functional output of the microbiota–immune–metabolism axis: SCFAs and some bile acids tend to maintain anti-inflammatory and metabolic homeostasis, lactic acid supports the local barrier of the reproductive tract, and the indole–AhR axis plays a pivotal role in barrier integrity, mucosal immunity, and inflammatory balance.

In summary, gut microbial metabolites participate in regulating the local immune environment and cellular metabolic processes of endometriosis through multiple mechanisms. SCFAs, as anti-inflammatory molecules, inhibit the proliferation and inflammation of ectopic endometrial lesions; bile acid metabolites regulate endocrine and immune responses; and indole metabolites maintain immune homeostasis through the AhR pathway (82). Simultaneously, AhR may further influence the persistence and complexity of the EMS lesion microenvironment through interactions with TGF-β-related immune/fibrotic signaling and antioxidant response pathways. The dynamic changes in these metabolites not only reflect the complex interactions between the gut and reproductive tract microenvironments but also provide potential metabolic biomarkers and intervention targets for the diagnosis and treatment of endometriosis.

### 3.3 Estrogen-microbiome (estrobolome) axis

The gut microbiota regulates the metabolism of estrogens through its unique enzyme systems, particularly β-glucosidase, and this microbial gene set is referred to as the “estrobolome” (83). These enzymes can deconjugate bound estrogens, thereby reactivating them and promoting their re-entry into the blood circulation via the enterohepatic circulation, influencing the levels of circulating estrogens in the body. Fluctuations in estrogen levels significantly impact the pathogenesis of hormone-dependent diseases, such as EMS (84). In EMS pathology, gut microbiota dysbiosis is characterized by a reduction in beneficial bacteria (e.g., Lactobacillus, Bifidobacterium, and Ruminococcaceae) and an expansion of pro-inflammatory bacteria (e.g., Escherichia, Shigella, Streptococcus, and Bacteroides) (84). This dysbiosis may lead to impaired intestinal barrier function, increased LPS release, and activation of the TLR4/NF-κB signaling pathway, thereby triggering systemic inflammatory responses and exacerbating the inflammatory state of EMS (85, 86). Simultaneously, the gut microbiota regulates estrogen levels through the estrobolome, affecting lesion proliferation and disease progression (87). Although studies report no significant differences in β-glucosidase activity, microbial diversity, or overall abundance in the gut microbiota of patients with EMS, specific taxa such as *Erysipelotrichia* are significantly enriched (88). This enrichment coincides with elevated levels of related estrogens and their metabolites, suggesting a complex association between the gut microbiota and estrogen metabolism. Furthermore, changes in the function of the intestinal estrobolome may influence the hormone-dependent pathology of EMS by modulating estrogen recirculation, serving as a potential diagnostic and therapeutic target (89).

In addition to the intestinal estrobolome, there is also bidirectional regulation between the local microbiota of the reproductive tract and estrogen signaling. Estrogen can promote glycogen deposition in the vaginal epithelium, providing a substrate for Lactobacillus colonization, thereby supporting a low-inflammatory ecology dominated by “Lactobacillus.” Conversely, when Lactobacillus decreases and anaerobic bacteria such as *Gardnerella, Atopobium*, and *Prevotella* increase, local pH, mucosal barrier function, and inflammatory status may all change, subsequently affecting estrogen receptor signaling, local immune responses, and the lesion microenvironment (90–92). In other words, the estrogen–microbiota interaction in EMs is not only reflected in abnormal intestinal estrogen recirculation but may also involve the regulation of hormone sensitivity and inflammatory thresholds by the local microecology of the reproductive tract.

The composition and function of the gut microbiota are influenced by various factors, including age, obesity, menopausal status, and dietary habits. For example, the decline in estrogen levels after menopause is associated with reduced gut microbiota diversity, an increased *Firmicutes/Bacteroidetes ratio*, and altered β-glucosidase activity of the estrobolome, leading to abnormal systemic estrogen levels and an increased risk of endometrial cancer and other estrogen-related diseases (93, 94). Moreover, the gut microbiota can metabolize dietary phytoestrogens, producing more active metabolites such as equol, which further influence estrogen receptor signaling pathways, potentially impacting hormone regulation in EMS. The interaction between the gut microbiota and estrogen metabolism not only affects local lesions but may also contribute to the pathogenesis and progression of EMS by modulating systemic inflammation and immune status (95). For instance, gut microbiota-mediated dysregulation of inflammatory factors and immune cell functions promotes the maintenance of a chronic inflammatory environment in EMS lesions, facilitating lesion survival and spread (96). Based on research on the estrobolome, future interventions targeting estrogen metabolism abnormalities in endometriosis may be achieved by modulating the gut microbiota. For example, methods such as probiotics, prebiotics, or fecal microbiota transplantation (FMT) may restore gut microecological balance, reduce β-glucosidase activity, and thereby regulate estrogen levels and alleviate EMs symptoms (97, 98). Additionally, emerging metabolomics and transcriptomics technologies will help further elucidate the molecular mechanisms of the gut microbiota–estrogen axis, providing a basis for precision treatment (99).

In summary, the estrobolome axis encompasses the combined outcome of gut microbiota-mediated estrogen recirculation and the interaction between the local microecology of the reproductive tract and hormones. The former primarily influences circulating estrogen load, while the latter largely determines the local mucosal environment, inflammatory thresholds, and lesion responsiveness to hormonal stimulation. Together, they contribute to the occurrence and development of EMS, a hormone-dependent disease.

### 3.4 Inflammation-coagulation-metabolism network

The interaction between the inflammatory response and the coagulation system constitutes a crucial component of the complex regulatory network within the microenvironment of EMS lesions, thereby driving metabolic remodeling and pathological progression of the lesions (100). Multiple studies have shown that EMS patients exhibit a significant chronic inflammatory state accompanied by abnormal activation of the coagulation system. This interplay between inflammation and coagulation not only promotes local tissue fibrosis and neovascularization but also triggers profound alterations in metabolic pathways, forming a dynamic pathological network (101, 102).

First, from the perspective of inflammation, the infiltration of inflammatory cells and the release of pro-inflammatory factors, such as cytokines and chemokines, within EMS lesions activate local immune responses and promote inflammatory cascades (103). Studies have found that components of the complement system (e.g., C3, C7) and serum protease inhibitors (e.g., SERPINE1, SERPINE2) are significantly upregulated in EMS lesions and related plasma, indicating the active involvement of complement and coagulation cascades (104, 105). Complement activation not only directly amplifies inflammation but also interacts with the coagulation system by promoting platelet activation and fibrin deposition, forming a vicious cycle of pro-inflammatory and pro-coagulant effects (106).

Second, the abnormal activation of the coagulation system in EMs is manifested by increased expression of tissue factor (TF) and elevated levels of circulating microparticles (cMPs), particularly more pronounced in patients with deep infiltrating endometriosis (DIE). Circulating microparticles carrying tissue factor (cMP-TF) not only enhance local coagulation responses but also regulate inflammatory reactions by activating downstream signaling pathways, creating a positive feedback loop between inflammation and coagulation (107). Additionally, hormonal therapies, such as continuous combined estrogen-progestogen oral contraceptives, can modulate cMP and cMP-TF levels, reflecting the dynamic plasticity and therapeutic potential of the inflammation-coagulation network.

**Figure 2:**
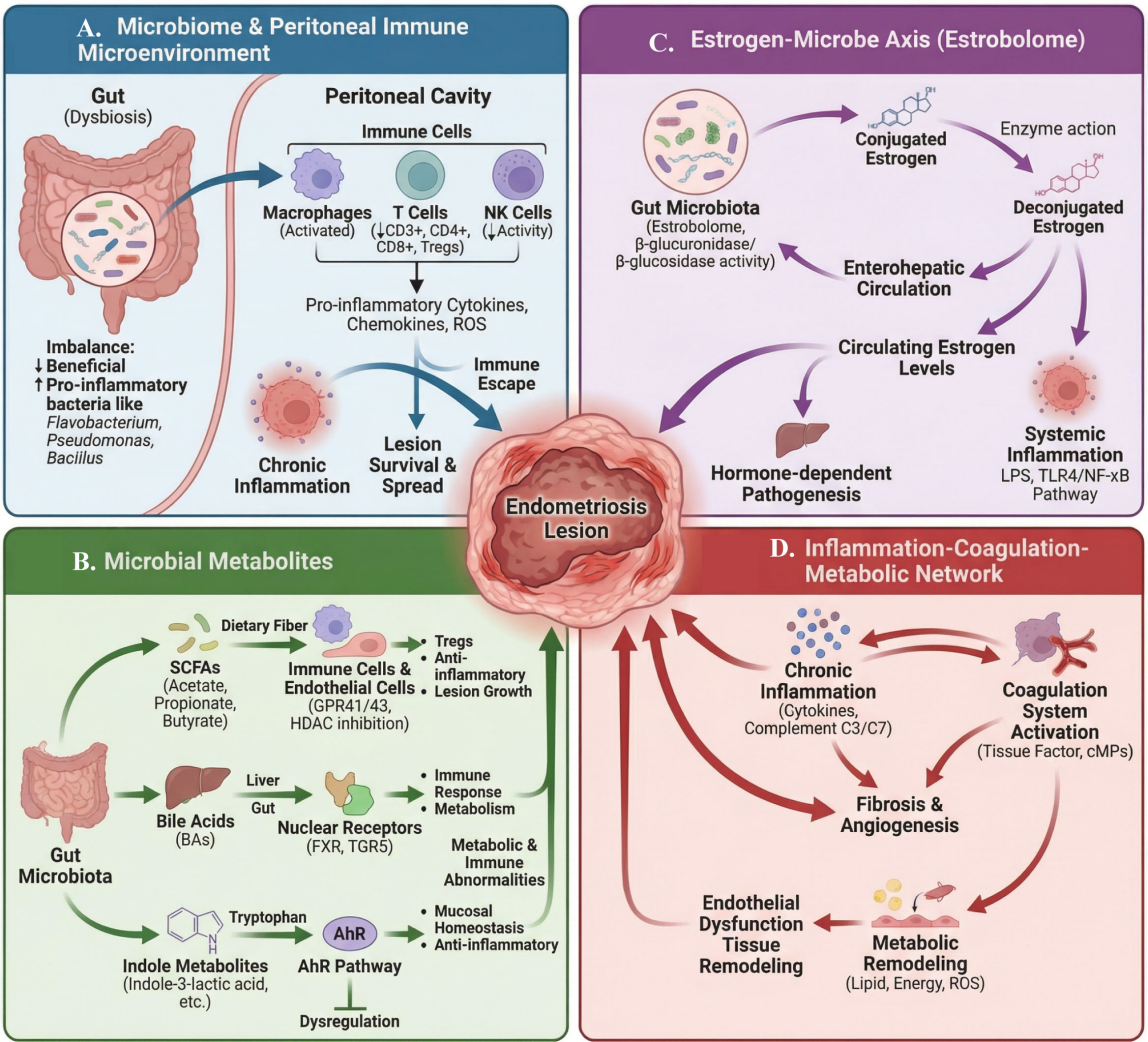
Gut-reproductive tract microbiota influences EMS through immune and metabolic pathways: **(A)** dysbiosis of gut microbiota can activate peritoneal macrophages, T cells, and NK cells, producing inflammatory cytokines and ROS, leading to chronic inflammation and promoting lesion survival and immune evasion; **(B)** gut-derived short-chain fatty acids, bile acids, and indole metabolites regulate mucosal immunity and metabolic homeostasis through the GPCR, nuclear receptor, and AhR pathways, promoting lesion growth when imbalanced; **(C)** the gut “estrogen microbiota (estrobolome)” regulates the estrogen enterohepatic circulation through β-glucosidase, increasing circulating estrogen and activating LPS-TLR4/NF-κB–related inflammation, exacerbating hormone-dependent disease progression; **(D)** the components of the inflammation-coagulation-metabolism network amplify one another, with chronic inflammation driving coagulation system activation, fibrosis, and angiogenesis, accompanied by lipid and energy metabolism remodeling, ultimately promoting the progression and recurrence of endometriotic lesions. (BAs, bile acids; AhR, Aryl hydrocarbon receptor; FXR, Farnesoid X receptor; ROS, reactive oxygen species). This image was drawn using BioRender software (https://app.biorender.com).

Metabolic remodeling is a key pathological feature driven by the inflammation-coagulation network. Abnormal expression of proteins related to lipid metabolism, energy metabolism, and oxidative stress in EMS lesions reflects profound reprogramming of metabolic pathways. For instance, proteomic analyses have shown upregulation of proteins associated with lipid transport, cell migration, and oxidative damage, while proteins involved in cell junctions, metabolism, and energy responses are downregulated, suggesting that inflammation and coagulation activation drive cellular metabolism toward a pro-inflammatory phenotype (108). Metabolic remodeling not only provides the energy and biosynthetic precursors required for sustained inflammation in lesions but also enhances inflammatory signaling through the generation of ROS and lipid peroxidation products (e.g., 4-hydroxy-2-nonenal), promoting endothelial dysfunction and local tissue remodeling (109). Furthermore, studies on gene expression and signaling pathways reveal that the inflammation-coagulation network is closely linked to extracellular matrix (ECM) remodeling and epithelial-mesenchymal transition (EMT). Inflammatory factors and activated coagulation cascades promote the expression of ECM components such as fibronectin (FN1) and collagen, facilitating the adhesion and invasive growth of lesion tissues (110, 111). Additionally, transcription factors such as KLF2 and HOXB6 are involved in regulating immune and metabolism-related genes, indicating that inflammation-coagulation signals modulate lesion metabolism and the immune microenvironment at the transcriptional level (112) (**Figure 2**). In summary, inflammation and the coagulation system are tightly intertwined within EMs lesions, forming a complex signaling network. This network not only drives local inflammatory responses and coagulation processes but also triggers the remodeling of metabolic pathways, promoting the onset and progression of the disease. Inflammatory factors induce coagulation cascades, while coagulation factors, in turn, enhance inflammatory signals. Together, they act on cellular metabolism and tissue structure, forming a vicious cycle of pro-inflammatory, pro-coagulant, and metabolic abnormalities.

In conclusion, the dysbiosis in EMS should be understood from the perspective of the “gut-reproductive tract” dual ecological niche. Gut microbiota imbalance can affect systemic immune and metabolic homeostasis through SCFAs, bile acids, indole metabolites, and estrogen recycling, while local dysbiosis in the reproductive tract and peritoneal cavity more directly contributes to barrier disruption, inflammation amplification, and immune evasion. Notably, the AhR not only mediates microbial metabolic signal transduction but may also integrate signals related to indoleamine IDO1–kynurenine metabolism, inflammation, oxidative stress, and TGF-β, serving as a critical regulatory node for immune-metabolic abnormalities in EMS. Furthermore, the intertwining of inflammation and coagulation networks drives energy and lipid metabolic reprogramming, creating an abnormal microenvironment characterized by elevated ROS levels and lipid peroxidation. This is coupled with disturbances in iron homeostasis and susceptibility to ferroptosis, collectively forming the essential foundation of the “gut-reproductive tract microbiota–iron metabolism–ferroptosis” mechanistic chain.

## 4 Imbalance of iron metabolism and iron overload environment in EMS

The biochemical chain of “retrograde menstruation/recurrent bleeding leading to local iron-rich—oxidative stress—lipid peroxidation” has a relatively well-established mechanistic basis; however, when it is further organized into a causal cascade of “gut microbiota imbalance→iron metabolism disorder→ferroptosis→lesion progression and infertility,” it still necessitates a nuanced interpretation that considers the source and strength of evidence. Specifically, it is important to differentiate among EMS-specific human evidence (patient body fluid/tissue testing and clinical relevance analysis), EMS-specific mechanistic evidence (functional intervention studies in cells from lesion sources and animal models), and mechanistic evidence transferable to non-EMS scenarios (such as tumors or other inflammation/metabolism models). Among the existing evidence, the most robust link is “iron overload/iron homeostasis abnormality→enhanced lipid peroxidation and altered susceptibility to ferroptosis”; whereas human causal evidence in EMS linking “ferroptosis→lesion progression and infertility, representing clinical hard endpoints” remains limited and is currently primarily derived from *in vitro* and animal studies, the “gut microbiota imbalance→iron metabolism disorder” connection in EMS is still mainly based on correlational observations and extrapolation across diseases.

### 4.1 Recurrent bleeding during menstruation and pelvic iron accumulation

Repeated bleeding during menstruation and retrograde menstrual flow constitutes a key pathological basis for EMS, and this process is closely related to the local deposition of iron ions in the pelvic cavity and the formation of an iron-rich microenvironment (113). The menstrual blood contains a large amount of hemoglobin and iron; under normal circumstances, menstrual blood is expelled from the body. However, in EMS patients, menstrual blood flows back into the pelvic cavity and accumulates in the pelvic cavity and surrounding ectopic endometrium, causing iron ions to gradually deposit with repeated bleeding, leading to local iron overload (114). Currently, observational studies demonstrate that the iron levels in the pelvic fluid and lesion microenvironment of EMS patients are elevated and correlated with the severity of the disease, providing EMS-specific human-related evidence for the “menstrual blood-related iron-rich microenvironment.”

At the mechanistic level, free iron can promote the generation of ROS through the Fenton reaction and amplify oxidative stress, thereby enhancing lipid peroxidation and providing a biochemical basis for iron-dependent regulated cell death (ferroptosis) (115). It is important to emphasize that although the biochemical chain mechanism of “iron-rich → oxidative stress/lipid peroxidation” is clear, at the human level of EMS, whether the “iron-rich state necessarily initiates a complete and repeatable ferroptosis molecular program (such as downregulation of GPX4/SLC7A11, upregulation of ACSL4, and accumulation of lipid peroxides)” still lacks systematic causal verification; at this stage, it relies more on small sample lesion biomarker indications and *in vitro*/animal experimental consistency support, thus it is more appropriate to express it as a “potential causal pathway with high biological plausibility,” rather than a closed-loop proof completed by human studies (116). Furthermore, iron overload-related oxidative damage may affect the function of local immune cells and weaken immune clearance, thereby providing conditions for the survival and spread of ectopic endometrium (117). At the same time, the iron-rich environment may also affect reproductive cells such as ovarian granulosa cells, increasing the risk of ferroptosis-like cellular damage and affecting fertility (118). However, these “downstream clinical consequences” (persistent lesions/fibrosis or impaired reproductive outcomes) in the human body of EMS still mainly belong to correlation and indirect evidence, lacking sufficient longitudinal follow-up or interventional studies to determine directionality and causality.

In addition, some studies suggest that changes in gut microbiota are associated with iron metabolism disorders, and certain bacteria can affect host iron absorption and iron homeostasis, potentially participating in iron load regulation (119). However, it should be clarified that in the EMS scenario, the evidence for “gut microbiota imbalance → iron metabolism disorder/pelvic iron load changes” is currently weak, mostly derived from cross-disease inferences or general mechanistic extrapolations, lacking EMS-specific longitudinal human studies (tracking changes in microbiota and iron indicators over time) or microecological intervention studies to prove directionality and causal chains. Therefore, this review considers it a promising upstream clue warranting further validation, rather than as an established causal connection.

### 4.2 Pelvic/ovarian local iron homeostasis regulation abnormalities

Abnormal regulation of local iron homeostasis in the pelvis and ovaries is considered to play a crucial role in the formation and maintenance of the iron-rich microenvironment in EMS (120, 121). Hepcidin is a core regulatory factor of systemic and local iron homeostasis, primarily through binding to the iron export protein ferroportin, inducing its endocytosis and degradation, thereby limiting iron efflux and affecting the intracellular free iron pool (122). In studies related to EMS, abnormalities in local hepcidin and iron transport-related molecules are associated with the accumulation of iron ions, oxidative stress, and enhanced lipid peroxidation, providing consistent mechanistic evidence for “iron homeostasis imbalance → altered susceptibility to ferroptosis” (12). However, current evidence at the patient level for EMS primarily focuses on differences in tissue/body fluid biomarkers and clinical relevance, and lacks functional causal validation targeting the hepcidin–FPN axis (such as upregulation/downregulation of the axis in lesion-derived cells or animal models, with endpoints of lesion burden, fibrosis, or reproductive outcomes). Therefore, it is more appropriate to position this axis as a key mechanistic module with testable causal potential rather than a causal hub that has been fully confirmed by clinical studies.

It is noteworthy that evidence regarding “increased iron uptake—enhanced iron storage—reduced iron output” leading to the expansion of intracellular iron pools and increased lipid peroxidation pressure, thereby altering sensitivity to ferroptosis, is more substantial in tumor and other inflammation/stress models. For example, the “iron addiction” phenotype in high-grade serous ovarian cancer (HGSOC), treatment-induced senescence-related iron accumulation, and the effects of iron chelation interventions on TfR1/ferritin and cell death outcomes provide important transferable clues for understanding how iron homeostasis components shape the threshold for ferroptosis (123–125). In EMs, the local regulation of hepcidin not only affects iron metabolism but is also closely related to redox signaling pathways, lipid metabolism, and mechanisms of cell death (126). For instance, overexpression of hepcidin can promote intracellular accumulation of iron, induce lipid peroxidation, and impair mitochondrial function, thereby activating ferroptosis (127). Studies on the correlation between iron homeostasis disorder and aging in ovarian tissue also indicate that abnormalities in hepcidin and its regulated iron transport proteins are the root cause of local iron overload and oxidative damage. Iron chelators such as deferoxamine (DFO) can effectively improve this condition and delay the decline of ovarian reserve (128). Moreover, the abnormal expression of hepcidin and iron transport proteins is also closely related to the drug resistance of cancer cells. For example, SGK1 maintains intracellular iron homeostasis and inhibits ferroptosis by regulating Nrf2-dependent and -independent pathways, thereby increasing tolerance to chemotherapy (129). Blocking the function of hepcidin or its downstream iron transport proteins is expected to improve treatment outcomes by inducing ferroptosis and clearing residual tumor cells (130). However, since the above evidence primarily comes from non-EMs scenarios, caution is needed when extrapolating to EMS. These studies suggest that hepcidin, TfR1, ferritin, and FPN may constitute potential intervention nodes. However, their directional effects and causal contributions in the pelvic/ovarian microenvironment of EMS still require further validation through multi-omics integration and functional experiments using EMS patient samples.

In summary, there is a clear mechanistic coupling basis between abnormal pelvic/ovarian iron homeostasis and lipid peroxidation as well as susceptibility to ferroptosis; however, from the perspective of the “causal cascade” framework, the current evidence structure for this link is more consistent with the state of “mechanistic support is relatively sufficient, human-level evidence is primarily correlational, and causal validation is insufficient.”

### 4.3 The impact of iron overload on ovarian function and oocyte quality

Iron overload is widely recognized as an important pathological factor leading to ovarian dysfunction and oocyte quality decline in EMS and other gynecological diseases. Excessive accumulation of iron can induce cell damage through various mechanisms, especially by inducing oxidative stress and ferroptosis pathways, severely damaging ovarian tissues and oocytes, thereby affecting female fertility (131). First, iron overload promotes the production of ROS, triggering lipid peroxidation reactions, causing cell membrane damage and mitochondrial dysfunction (132). Studies have shown that iron overload significantly increases the levels of lipid peroxidation products, malondialdehyde (MDA), in the ovaries, while the expression of antioxidant enzymes such as glutathione peroxidase 4 (GPX4) and glutathione (GSH) is downregulated, indicating a decline in antioxidant defense capacity, which exacerbates oxidative stress (133). This oxidative environment not only damages granulosa cells but also hinders oocyte maturation, manifested as a reduced polar body extrusion rate and decreased embryonic developmental potential (134). Secondly, iron overload also affects iron homeostasis by regulating the expression of iron metabolism-related proteins. Excess iron deposits locally in the ovaries, leading to the activation of iron-dependent cell death—ferroptosis, accompanied by cellular pathological changes such as decreased mitochondrial membrane potential, increased apoptosis, mitochondrial hyperfission, and endoplasmic reticulum stress (135, 136). Ferroptosis markers such as ACSL4 and COX2 are upregulated, while SLC7A11 and GPX4 are significantly downregulated, revealing that ferroptosis induced by iron overload plays a key role in ovarian granulosa cells (137, 138). The evidence from *in vitro* and animal experiments can provide directional mechanistic support for “iron overload → ferroptosis-like damage → decline in ovarian cell function,” but caution is needed when extrapolating this to fertility outcomes in EMS patients.

In addition, iron overload can disrupt ovarian endocrine function and reduce estrogen synthesis. Research has found that iron overload inhibits granulosa cell function through the ROS-mediated HIF-1α/FSHR/CYP19A1 signaling pathway, leading to decreased estrogen synthesis, which in turn causes abnormal oocyte development and decreased fertility (139). Clinically, the R2 value of magnetic resonance imaging (MRI) can reflect the degree of iron deposition in the ovaries and is negatively correlated with the level of anti-Müllerian hormone (AMH), suggesting that iron overload is closely related to the decline in ovarian reserve function (140). Ferroptosis induced by iron overload not only damages granulosa cells but also further inhibits oocyte maturation by releasing extracellular vesicles, exacerbating ovarian dysfunction (141). However, there is still a lack of sufficient prospective cohort or intervention studies to address the directional question of whether “iron overload/ferroptosis is a causal driving factor or key mediator of infertility.” Therefore, a more evidence-based statement should be: iron overload and ferroptosis provide a highly interpretable mechanistic framework for EMS-related reproductive damage, but “ferroptosis → infertility” remains in the evidence accumulation stage at the population level.

In chemotherapy-induced ovarian damage, the accumulation of iron ions triggers ferroptosis in granulosa cells, exacerbating the loss of ovarian reserve. Anti-ferroptosis treatments such as deferoxamine nanoparticles have been shown to effectively protect ovarian function (142, 143). Finally, in terms of intervention strategies, traditional Chinese medicine formulas, antioxidants (such as vitamin E and melatonin), iron chelators, and stem cell therapies have all shown potential to inhibit oxidative stress and ferroptosis, improving ovarian function (144). For example, the traditional Chinese medicine formula Huatuo detoxification can inhibit ferroptosis through the IL-6/angiotensin pathway, protecting ovarian function (145); melatonin can regulate ferroptosis and mitochondrial function in granulosa cells, enhancing IVF success rates (144); HF-MSCs intervention can inhibit ferroptosis in granulosa cells through the KEAP1/NRF2/HO-1 signaling pathway, promoting the recovery of ovarian function (146). However, most current evidence is preclinical or from non-EMs scenarios, and given the reproductive safety and long-term management needs involved, it is advisable to position these as “mechanism-driven candidate directions” rather than directly recommendable proximal clinical strategies.

### 4.4 Iron overload and oxidative stress, lipid peroxidation

**Figure 3:**
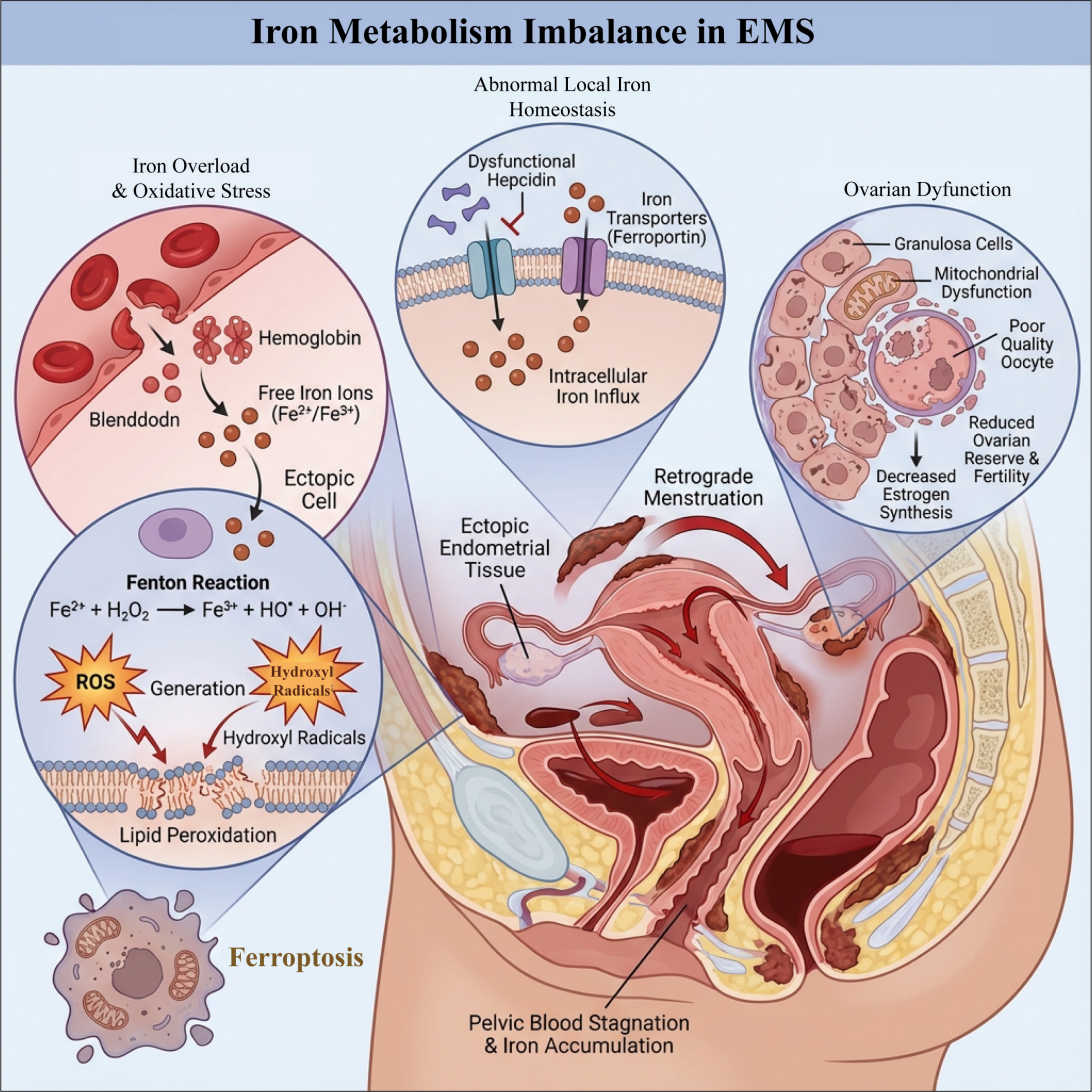
Schematic diagram of iron metabolism imbalance in endometriosis. Retrograde menstruation and pelvic hemorrhage lead to local iron overload in ectopic lesions, where red blood cell lysis releases Fe²^+^/Fe³^+^. These ions produce a large amount of ROS and hydroxyl radicals through the Fenton reaction, triggering lipid peroxidation and ferroptosis. The abnormal function of hepcidin/iron transport proteins in the lesion area further promotes intracellular iron accumulation. Additionally, long-term iron load and oxidative stress damage ovarian granulosa cells and mitochondria, reducing oocyte quality, the synthesis of estrogen, and ovarian reserve, ultimately affecting fertility. This image was drawn using BioRender software (https://app.biorender.com).

Iron overload refers to the abnormal accumulation of free iron ions in the body, which can catalyze the generation of ROS, leading to lipid peroxidation and cell damage. This process plays a central role in the formation of EMS lesions. First, iron, as a catalyst for the Fenton reaction, promotes the conversion of hydrogen peroxide into hydroxyl radicals, resulting in a large amount of ROS production (147). These excess ROS not only damage cellular proteins and DNA but also directly attack polyunsaturated fatty acids on cell membranes, causing lipid peroxidation and disrupting membrane structure and function (148, 149). Studies have shown that iron overload significantly increases mitochondrial ROS production by inducing mitochondrial iron accumulation. This leads to a decrease in mitochondrial membrane potential and mitochondrial dysfunction, ultimately triggering an iron-dependent form of programmed cell death known as ferroptosis (150). The typical characteristics of ferroptosis include reduced activity of GPX4 and accumulation of lipid peroxides, which are also observed in EMs lesion cells. Iron-mediated ROS exacerbate lipid peroxidation by downregulating GPX4 and altering the expression of iron homeostasis-related proteins such as heme oxygenase-1 (HO-1) and BTB and CNC homology 1 (Bach1). These changes contribute to the damage of cell membrane integrity (151, 152). In addition, iron overload can affect phospholipid metabolism, especially the metabolic pathways of arachidonic acid (ARA). Oxidative stress caused by iron overload reduces the levels of ARA metabolites such as 12S-hydroxyeicosatetraenoic acid and 15S-HETE, disrupting the cyclooxygenase (COX) and lipoxygenase (LOX) metabolic pathways of ARA. This disruption affects inflammatory responses and cell signaling (153). Iron-induced lipid peroxidation not only damages cell membranes but also releases pro-inflammatory mediators. These mediators promote the formation of a chronic inflammatory environment in lesions, thereby exacerbating the pathological progression of EMS (154). Multiple animal and cell studies have demonstrated that antioxidants and iron chelating agents can alleviate oxidative damage caused by iron overload, inhibit lipid peroxidation and ferroptosis, improve tissue function, and reduce pathological changes (155). For example, naringenin reduces ROS and lipid peroxidation induced by iron overload and alleviates tissue damage by activating the nuclear factor erythroid 2-NRF2-HO-1 antioxidant pathway (156). Iron chelators such as DFO reduce free iron levels, blocking the iron-mediated lipid peroxidation chain reaction and protecting cells from ferroptosis (157) (**Figure 3**). In summary, iron overload catalyzes the generation of ROS, induces lipid peroxidation and ferroptosis, constituting a key pathological mechanism in the formation of EMS lesions.

## 5 The dual role of ferroptosis in EMS

### 5.1 Basic concept and core pathway of ferroptosis

Ferroptosis is a newly discovered form of programmed cell death characterized by the accumulation of iron-dependent lipid peroxides, distinct from traditional mechanisms of cell death such as apoptosis and necrosis (158). Ferroptosis was first proposed by Stockwell et al. in 2012 and has recently become a hotspot in the study of cell biology and disease pathology, involving multiple fields such as tumors, neurodegenerative diseases, cardiovascular diseases, autoimmune diseases, and metabolic diseases (159, 160). The core features of ferroptosis include: (1) intracellular iron ion overload, with iron generating a large amount of ROS through the Fenton reaction; (2) enhanced lipid peroxidation, especially the oxidation of polyunsaturated fatty acid (PUFA) phospholipids, causing cell membrane damage; (3) decreased GPX4 activity, resulting in weakened antioxidant defense capability; (4) mitochondrial morphological changes, including volume shrinkage, increased membrane density, and reduced cristae (161, 162). At the molecular regulatory level, GPX4 is considered a key inhibitory factor of ferroptosis. GPX4 uses reduced glutathione (GSH) to convert lipid peroxides into non-toxic lipid alcohols, thereby preventing their accumulation (163). When the function of system X_c (cystine/glutamate antiporter) is impaired, intracellular cysteine supply decreases, leading to reduced GSH synthesis and decreased GPX4 activity, thus initiating ferroptosis. In addition, increased influx of iron ions and abnormal fatty acid metabolism also promote lipid peroxidation (164). The transcription factor NRF2 (protein encoded by the NFE2L2 gene) also plays an important role in the regulation of ferroptosis. NRF2 enhances cellular antioxidant capacity and inhibits ferroptosis by regulating the expression of antioxidant genes, such as SLC7A11, a subunit of system Xc, and GPX4. Moreover, NRF2 can regulate iron homeostasis-related proteins, reducing iron ion accumulation and thus slowing down the process of ferroptosis (165, 166). Studies have shown that NFE2L1 (NRF1) can also regulate GPX4 expression independently of NRF2, contributing to ferroptosis resistance (167). The occurrence of ferroptosis involves the intertwined regulation of multiple signaling pathways. For example, there is the interaction between the Hippo pathway and ferroptosis in regulating cell fate (168); the MAPK pathway regulates the expression of ferroptosis-related genes (169); and inflammatory signaling pathways such as JAK-STAT and NF-κB participate in the regulation of ferroptosis (170). In addition, mitochondrial dysfunction, reprogramming of lipid metabolism, and post-translational ubiquitination modifications within cells also have significant implications for the regulation of ferroptosis (171, 172). In summary, ferroptosis, as an iron-dependent non-apoptotic form of cell death, has unique biochemical characteristics and a complex regulatory network. Key regulatory molecules include GPX4, NRF2, and their upstream and downstream molecules, which regulate iron homeostasis, antioxidant defense, and lipid metabolism.

### 5.2 Ferroptosis of endometrial stromal cells and lesion progression

The presence of iron overload and oxidative stress in EMS lesion tissues establishes the biochemical prerequisites for ferroptosis. However, the specific role of ferroptosis in lesion cells exhibits a dual nature, promoting both lesion cell death and contributing to lesion progression and fibrosis processes (116, 173).

It is important to emphasize that this “double-edged sword” effect is not accidental but primarily depends on the cell type in which ferroptosis occurs, the intensity of induction, the microenvironmental context, and the cell’s own anti-ferroptosis buffering capacity. In ectopic endometrial stromal cells (ESCs) residing within a lesion microenvironment characterized by iron overload, elevated ROS, hypoxia, and chronic inflammation, lipid peroxidation pressure that fails to reach the “lethal threshold” often induces a state of “sublethal ferroptotic stress” (174). In this state, the cells are not effectively cleared; instead, they can release pro-angiogenic and pro-inflammatory factors such as vascular endothelial growth factor A (VEGFA) and IL-8, inducing activation of adjacent endothelial cells, promoting EMT and fibrotic remodeling, thereby driving lesion maintenance and expansion (175, 176). Conversely, if lipid peroxidation, free iron accumulation, and antioxidant system collapse reach a level sufficient to trigger irreversible cell death, ferroptosis is more likely to manifest as a “lesion clearance effect,” reducing the load of viable ectopic cells (177, 178). In other words, the key determinant of whether ferroptosis has a harmful or beneficial outcome is not merely ‘whether ferroptosis occurs,’ but rather ‘in which cells, in which microenvironment, and at what intensity it occurs.’

First, studies have found that some ESCs in ovarian endometrial implantation cysts are affected by ferroptosis and undergo death. Ferroptosis triggers these cells to secrete various pro-angiogenic, pro-inflammatory, and pro-growth cytokines, such as VEGFA and IL-8, promoting angiogenesis in adjacent vascular endothelial cells through paracrine action, thereby driving lesion expansion and maintenance (179). This indicates that ferroptosis does not simply lead to cell death and lesion reduction but promotes lesion environment remodeling and progression by regulating cytokine release. On the other hand, ferroptosis also plays an important role in fibrogenesis within lesions. Iron accumulation in lesion tissues increases lipid peroxidation and oxidative stress levels, activating stromal cell transformation and fibrosis processes. Experimental evidence shows that inducing ferroptosis in ESCs can promote the expression of EMT and fibrotic markers, facilitating fibrogenesis in lesions (180, 181). Furthermore, ferroptosis inhibitors such as ferrostatin-1 can significantly alleviate endometrial fibrosis, suggesting that regulating ferroptosis has potential therapeutic value. Furthermore, ferroptosis is closely related to cellular signaling pathways within lesions (182). For example, the p38 MAPK/STAT6 signaling pathway regulates the expression of pro-angiogenic factors induced by ferroptosis, and METTL3-mediated m6A modification inhibits ferroptosis in ESCs by regulating HMOX1 expression, thereby promoting lesion repair and fibrosis (183). EZH2, as an inhibitor of ferroptosis, plays a key role in regulating the sensitivity of ESCs to ferroptosis (184). During disease progression, ESCs exhibit tolerance to ferroptosis, partly dependent on the regulation of intracellular antioxidant systems, such as the expression of GPX4 and its regulation by m6A modification (185, 186). Therefore, mechanistically, the divergent outcomes of ferroptosis within lesions are determined by at least the following factors: (1) whether lesion cells are already in a “ferroptosis-susceptible” state characterized by high iron/high ROS/high polyunsaturated fatty acid (PUFA) load; (2) whether anti-ferroptosis axes such as GPX4, SLC7A11, NRF2, FSP1, ATF4-xCT can still maintain lipid peroxidation buffering; (3) whether ferroptosis is a focal and sufficient “lethal strike” or an insufficient “sublethal stress” that fails to clear cells; (4) whether the surrounding stroma, inflammatory cells, and vascular components further transform this stress into pro-inflammatory, pro-angiogenic, and pro-fibrotic signals. It is in this sense that ferroptosis manifests as a context-dependent double-edged sword in EMS lesions, rather than a simple “pro-disease” or “anti-disease” process.

**Figure 4:**
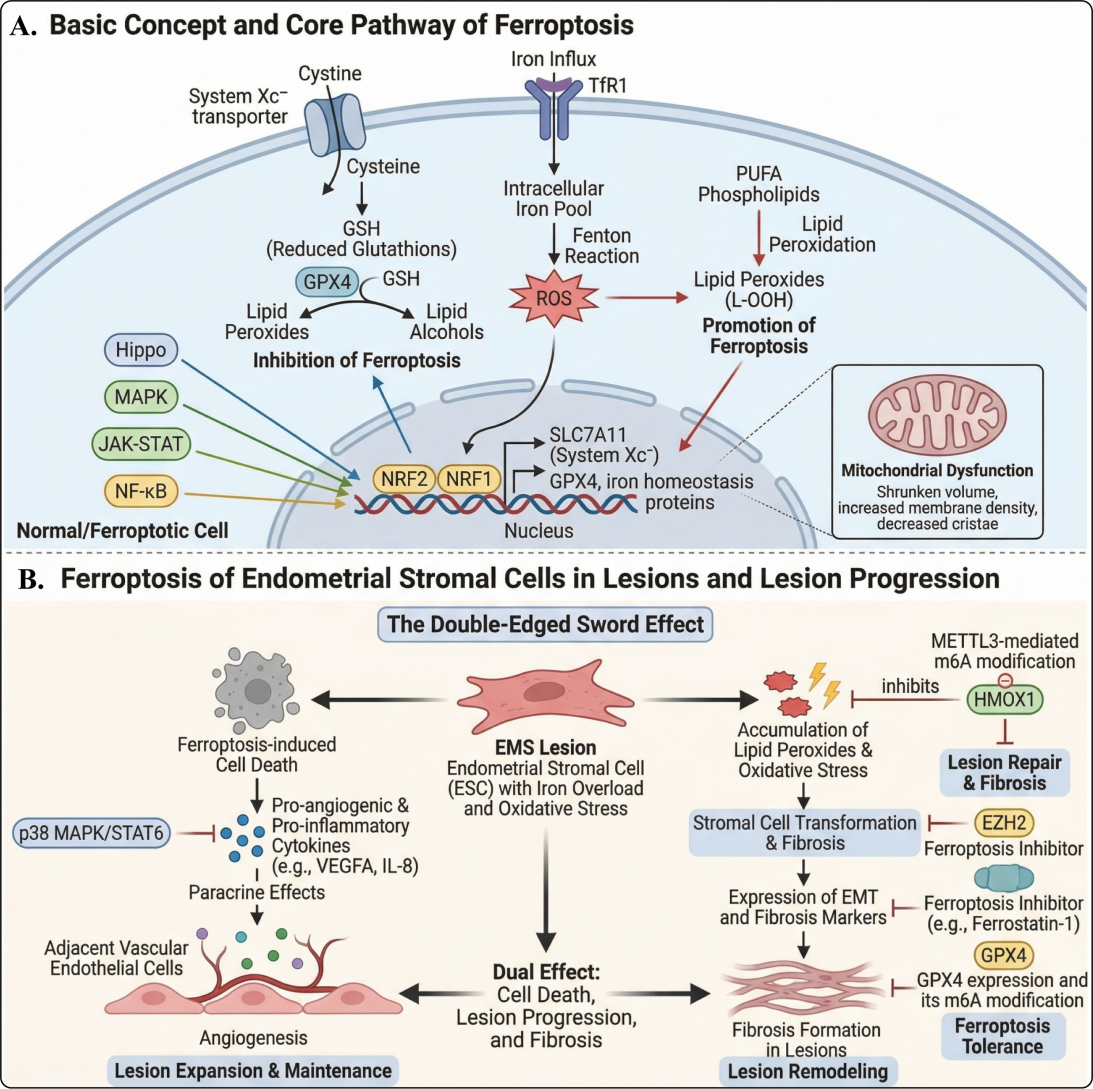
Core ferroptosis pathways and the double-edged role of ferroptosis in endometriotic lesions. **(A)** basic mechanism of ferroptosis: increased intracellular iron promotes ROS generation and lipid peroxide accumulation through the Fenton reaction, whereas the System Xc^-^–GSH–GPX4 axis suppresses ferroptosis by detoxifying lipid peroxides; NRF2/NRF1 and related signaling pathways participate in the regulation of antioxidant defense and iron homeostasis. **(B)** EMS lesions, iron overload and oxidative stress place stromal cells in a state where ferroptosis sensitivity and ferroptosis tolerance may coexist. On the one hand, ferroptotic cell death may be accompanied by release of pro-inflammatory and pro-angiogenic mediators that support lesion expansion and maintenance; on the other hand, lipid peroxidation, fibrotic transformation, and GPX4-associated ferroptosis tolerance may drive lesion remodeling and fibrosis. Overall, ferroptosis in EMS is not simply protective or detrimental, but rather a bidirectional regulatory process influencing lesion persistence, remodeling, and progression. (EMS, Endometriosis; ROS, reactive oxygen species; MAPK, Mitogen-activated protein kinase; NF-κB, Nuclear factor-κB; GPX4, Glutathione peroxidase 4; GSH, glutathione; VEGFA, Vascular endothelial growth factor A). This image was drawn using BioRender software (https://app.biorender.com).

In summary, ferroptosis in lesional endometrial stromal cells exerts a dual regulatory influence on the progression of EMS lesions. When ferroptosis reaches a sufficient intensity to surpass the cell clearance threshold, it predominantly results in a reduction of lesion cells; when it manifests as a localized, partial, or sublethal event, it may instead support lesion survival and expansion through the release of pro-inflammatory, pro-angiogenic, and pro-fibrotic signals. This also suggests that if ferroptosis is to be targeted therapeutically in the future, the strategic imperative lies not merely in “enhancing ferroptosis,” but in maximizing its lethality and specificity toward lesion cells, thereby preventing the sublethal states that drive lesion remodeling (**Figure 4**).

### 5.3 Ferroptosis of follicular/embryonic cells and infertility

Ferroptosis is characterized by the accumulation of iron-dependent lipid peroxidation and has recently been incorporated into the potential mechanism framework of female reproductive disorders. Clinically, the infertility rate among patients with EMS is relatively high (about 50%), with common phenotypes including abnormal follicular development and decreased oocyte quality (187). It is important to emphasize that when proposing “iron metabolism imbalance → ferroptosis → infertility” as a causal cascade, the following distinctions should be clearly made: (1) EMs-specific human evidence (the correlation between differences in patient body fluids/tissues and outcomes); (2) EMs-specific mechanistic evidence (patient sample-driven functional experiments, lesion-derived cell or animal model interventions); and (3) transferable clues from non-EMS scenarios (polycystic ovary syndrome (PCOS), chemotherapy, toxic exposure, aging models, etc.). Overall, in this section, the chain of “follicular microenvironment iron overload/increased oxidative stress” is relatively well-established in human EMs cases, while the human causal evidence for “ferroptosis as a key mediator leading to reproductive hard endpoints damage” remains limited.

At the level of EMS-specific human clues, multiple studies have observed that the follicular fluid (FF) of EMS patients exhibits increased iron levels, which tends to correlate with reproductive phenotypes such as oocyte maturation disorders (141). These findings support the notion that “iron metabolism abnormalities are associated with impaired follicular microenvironment,” but the type of evidence is mainly cross-sectional differences/correlations, which is insufficient to determine directionality and causality at the patient level. Mechanistically, existing studies further demonstrate a “testable causal potential”: FF from EMS patients can induce ferroptosis-related changes in mouse granulosa cells (GCs) *in vitro*, with key processes involving NCOA4-dependent ferritinophagy promoting the release of free iron and amplifying intracellular oxidative stress and lipid peroxidation damage (188, 189). Moreover, GCs undergoing ferroptosis-like damage may further inhibit oocyte maturation through paracrine mechanisms such as exosomes, thus forming an amplifying loop of “microenvironment deterioration - oocyte maturation obstruction” (190). Therefore, a more accurate statement reflecting the current evidence status is: functional experiments driven by EMS patient samples support the mechanistic consistency of “iron overload increasing GCs’ susceptibility to ferroptosis and impairing follicular function,” but “ferroptosis → EMS infertility (clinical hard endpoint)” still lacks sufficient human causal closure. Based on the aforementioned mechanistic consistency, some studies have suggested that antioxidants (such as vitamin E) and iron chelation strategies (such as Deferasirox) may alleviate iron overload-related lipid peroxidation and ferroptosis-like damage, thereby improving follicular function (191, 192). However, in the context of EMS, such recommendations should currently be positioned as “potential intervention strategies,” and their actual clinical effect size, safety profiles, and impacts on endpoints such as fertilization rates, embryo quality, and live birth still need to be validated within a standardized clinical trial framework.

Research in non-EMS scenarios provides “transferable biological support” for this chain, but it must also clearly define its extrapolation boundaries in the review. For example, in PCOS, the increased iron concentration in granulosa cells and lipid peroxidation products, along with the downregulation of GPX4 and upregulation of NCOA4, suggest that ferritinophagy and ferroptosis are activated (193). In androgen excess models, inhibiting ferroptosis (such as with ferrostatin-1) can improve ovarian morphology, ovulation, and enhance oocyte quality and embryo developmental potential (194). Environmental toxins (such as cadmium) exposure can induce oxidative stress/lipid peroxidation through mitochondrial dysfunction and GPX4 downregulation, and promote ferroptosis through autophagy-related mechanisms, thereby impairing follicular development (195). Chemotherapeutic agents (such as cisplatin and paclitaxel) can amplify lipid peroxidation through ROS and trigger ferroptosis, leading to decreased ovarian reserve and increased risk of primary ovarian insufficiency (POI), while antioxidant interventions like N-acetylcysteine (NAC) can reduce ROS and alleviate cell damage by enhancing GPX4, Nrf2, and HO-1 (143, 196). Additionally, in aging-related ovarian function decline, the downregulation of Cry1 and the enhancement of NCOA4-mediated ferritinophagy can promote ferroptosis and granulosa cell senescence, while the compound KL201, which stabilizes Cry1, can improve ovarian function by inhibiting ferritinophagy, suggesting that the “ferroptosis - aging - oocyte quality” axis is amenable to intervention (197). However, the aforementioned evidence mainly comes from non-EMs disease backgrounds or animal/cell models and should not be directly equated with EMs-specific human causal evidence. These findings can only serve as supporting clues for “mechanistic feasibility and potential intervention nodes,” and thus require further confirmation of their directionality and contribution in stratified EMs patient studies and functional validations.

In summary, existing evidence supports the notion that ferroptosis potentially damages within the follicle-embryo unit and can interfere with oocyte maturation and subsequent embryo development by disrupting granulosa cell homeostasis and exacerbating oxidative stress in the follicular microenvironment. However, regarding the “causal cascade,” the mechanistic rationale and model support are relatively strong, while EMS-specific human causal evidence and intervention evidence targeting reproductive hard endpoints remain relatively limited.

### 5.4 Ferroptosis and immune microenvironment remodeling

Ferroptosis not only directly affects the survival of tumor cells but also profoundly reshapes the functional state of immune cells, thereby regulating inflammatory and immune responses (198). First, ferroptosis influences the initiation and progression of inflammatory responses by regulating immune cell functions. In the tumor microenvironment, ferroptosis can induce the death of immunosuppressive cells, such as pro-tumor M2 tumor-associated macrophages (TAMs) and Tregs, thereby alleviating immunosuppression and promoting anti-tumor immune responses (199). For example, ferroptosis-induced death of M2 TAMs can promote their polarization to the anti-tumor M1 phenotype. This process activates cytotoxic T lymphocyte (CTL) infiltration, enhances immune cytotoxicity, and reshapes the tumor immune microenvironment (200). In addition, certain ferroptosis inducers can promote the maturation of dendritic cells (DCs), release tumor-associated antigens, and induce immunogenic cell death, further enhancing the body’s immune surveillance (201, 202). These changes in immune cell functions collectively enhance immune regulation, forming a microenvironment conducive to immune clearance. Second, ferroptosis may adjust the activation and infiltration status of immune cells by affecting their metabolism and signaling pathways. Different immune cells exhibit varying sensitivities to ferroptosis; for instance, CD8^+^ effector memory T cells show higher ferroptosis-related activity scores, with lipid peroxidation and ROS production during ferroptosis regulating their functions and differentiation (203, 204). Within the tumor microenvironment, ferroptosis-related genes regulate the infiltration patterns of immune cells, affecting the quantity and function of neutrophils, NK cells, dendritic cells, and T-cell subsets (205). Furthermore, the ferroptosis state regulates immunosuppression by modulating the expression of immune checkpoint genes (such as PD-1, CTLA4, and LAG3), thereby influencing the intensity and duration of inflammatory responses (206). Additionally, the interaction between ferroptosis and the immune microenvironment also involves mechanisms of immune tolerance and immune evasion. Damage-related molecular patterns (DAMPs) released by ferroptotic cells can activate immune responses; however, these cells may also release immunosuppressive factors through exosomes and other pathways, leading to immune tolerance (207, 208). This indicates that ferroptosis has a dual role in immune activation and immunosuppression, with specific effects depending on the microenvironment context and immune cell types. In pathological conditions such as EMS, ferroptosis influences the inflammatory microenvironment by regulating immune cell functions. Studies have shown that the expression of ferroptosis-related genes is closely related to the ratios of immune cell infiltration, such as M2 macrophages and regulatory T cells. Moreover, the regulation of ferroptosis can affect the degree of inflammatory responses and disease progression (11). At the same time, immune factors such as IL-33 protect endometriosis cells from ferroptosis by regulating the expression of the key ferroptosis molecule SLC7A11, thereby promoting disease progression. This indicates a close link between ferroptosis and immune regulation (209). In therapeutic aspects, nanotechnology has been widely utilized to regulate ferroptosis and the immune microenvironment. For example, iron-based nanoplatforms not only induce ferroptosis in tumor cells but also promote macrophage polarization, activate immune cell infiltration, and enhance the effects of immunotherapy (210, 211). Strategies combining immune checkpoint inhibitors have also shown synergistic enhancement of immune activation induced by ferroptosis (212). In summary, ferroptosis reshapes the inflammatory microenvironment by regulating the survival, polarization, and functions of immune cells, which can promote anti-tumor immunity but may also lead to immunosuppression and tolerance. Its dual role provides new insights into the pathological mechanisms of diseases such as EMS.

### 5.5 Estrogen, signaling pathways, and regulation of ferroptosis

Estrogen, as a key hormone in the female reproductive endocrine system, plays an important role in various physiological and pathological processes; it especially has a decisive impact on the occurrence and development of EMS. In recent years, ferroptosis, an iron-dependent regulated cell death mode, has gradually attracted attention for its interaction with the estrogen signaling pathway, providing a new perspective on the pathological mechanisms of EMS (213). Firstly, estrogen regulates cellular iron homeostasis and antioxidant capacity particularly through its receptor subtypes: estrogen receptor alpha (ERα), estrogen receptor beta (ERβ), and G protein-coupled estrogen receptor (GPER1), thereby affecting sensitivity to ferroptosis. Studies have shown that the absence of GPER1 leads to iron overload in microglia, promoting ferroptosis and exacerbating myelin damage; this indicates that estrogen receptor regulation of ferroptosis has a protective effect (214). In cardiac tissue, estrogen inhibits ferroptosis by upregulating the circadian rhythm protein Period 2 and related microRNAs, improving cardiac dysfunction under estrogen deficiency (215). However, contrasting with this protective effect, in male mouse models, chronic estrogen treatment induces oxidative stress and dysfunction related to myocardial ferroptosis, suggesting that the effects of estrogen are sex-dependent and tissue-specific (216). Secondly, estrogen modulates cellular resistance to ferroptosis by regulating key ferroptosis regulatory molecules such as SLC7A11, GPX4, and MBOAT1 (217). Estrogen receptor alpha upregulates the system Xc- components SLC7A11 and SLC3A2 in ER^+^ breast cancer cells, enhancing the cells’ resistance to ferroptosis and reducing sensitivity to endocrine therapy, suggesting that targeting the estrogen-regulated system Xc- may promote ferroptosis and improve therapeutic efficacy (218). Additionally, estrogen slows down ferroptosis in endothelial-like stromal cells by upregulating the expression of membrane-bound O-acyltransferase MBOAT1, thereby promoting the progression of EMs (219). In skeletal muscle atrophy models, the natural flavonoid rutin inhibits estrogen deficiency-induced ferroptosis by directly binding to SLC7A11, which alleviates muscle loss (220). Estrogen receptor beta inhibits ferroptosis in intestinal epithelial cells by promoting GPX4 transcription in colitis, thereby reducing disease inflammation (221). Furthermore, estrogen metabolites 4-hydroxyestrone and 4-hydroxyestradiol, as inhibitors of protein disulfide isomerase (PDI), can effectively block the PDI-mediated ferroptosis pathway, demonstrating a cell-protective effect independent of classical estrogen receptors and providing evidence for a new mechanism of estrogen metabolites in ferroptosis regulation (222–224). In the pathology of EMS, estrogen signaling regulates sensitivity to ferroptosis through multiple pathways. The overexpression of estrogen receptor beta in EMS lesions is associated with the anti-ferroptosis mechanism and coordinates inflammatory responses and cell survival. MBOAT1-mediated phospholipid remodeling contributes to the regulatory mechanisms of ferroptosis and is closely related to the modulation of estrogen receptors (225). Additionally, estrogen can influence ferroptosis by regulating the PI3K/AKT signaling pathway. Related studies indicate that gut metabolites such as butyrate inhibit ferroptosis through the PTEN/PI3K/AKT pathway, which is indirectly modulated by estrogen (226). The interaction between estrogen and ferroptosis has complex bidirectional regulatory characteristics. On the one hand, estrogen inhibits ferroptosis through receptor-mediated transcriptional regulation and metabolites, promoting cell survival and lesion development. On the other hand, in certain tissues or pathological states, estrogen may enhance ferroptosis, leading to tissue damage and functional abnormalities, such as detrimental effects in the male myocardium (227). This complexity suggests that the regulatory mechanisms of estrogen on ferroptosis need to be comprehensively considered based on tissue type, sex, and pathological environment. In summary, estrogen regulates the expression and activity of key ferroptosis molecules through its diverse receptors and downstream signaling pathways, affecting the sensitivity and tolerance of cells to ferroptosis. This regulation involves not only classical transcription factor mechanisms but also non-classical signal transduction and enzyme activity regulation mediated by metabolites. These findings provide new molecular mechanism explanations and therapeutic targets for the occurrence and development of EMS.

### 5.6 The “harmful and beneficial” duality of ferroptosis

The mechanism of ferroptosis primarily involves iron ions catalyzing lipid peroxidation reactions, leading to the accumulation of lipid peroxides in cell membranes and ultimately triggering cell death. In recent years, ferroptosis has been found not only to participate in the pathogenesis of various diseases but also to exhibit a typical “double-edged sword” effect: on one hand, it exerts protective effects by clearing abnormal or diseased cells; on the other hand, under specific circumstances, it can damage normal tissue cells, leading to pathological harm (228). However, in the context of EMS, simply summarizing ferroptosis as “both harmful and beneficial coexisting” remains insufficient. A more critical question concerns which contextual factors dictate its progression toward lesion clearance versus those that drive lesion progression or impair reproductive function (229, 230). Based on existing evidence, this divergence in outcomes is governed by at least four categories of factors: (1) Cell type—whether ferroptosis occurs in ectopic stromal/epithelial cells, granulosa cells, oocytes, or immune cells (231); (2) Microenvironment status: local iron load, ROS levels,PUFA substrate supply, severity of hypoxia, and degree of inflammation (232); (3) Intracellular buffering capacity: the ability of anti-ferroptosis networks (e.g., GPX4/SLC7A11, NRF2, FSP1, ATF4-xCT, and GCH1-BH4) to restrain lipid peroxidation (233, 234); (4) Exposure mode—whether the ferroptosis stimulus is a localized and sufficient lethal strike or a low-intensity, diffuse, sublethal, and sustained stress. In other words, the outcome is determined not by “whether ferroptosis exists” but by “in which cells, under what microenvironment, and at what intensity ferroptosis occurs” (235).

On one hand, in ectopic stromal and epithelial cells, as well as lesion epithelial cells, “moderate, focal” ferroptosis is considered to exert a relatively protective effect. EMs lesion tissues often exhibit excessive local iron load, and iron accumulation induces ferroptosis in ectopic endometrial cells, thereby limiting lesion expansion and development (13, 236). For example, β-elemene inhibits the proliferation and migration of ectopic endometrial stromal cells by inducing ferroptosis, slowing lesion growth (237); the ferroptosis inducer erastin promotes ferroptosis in ectopic endometrial cells both *in vitro* and *in vivo* and inhibits lesion formation (238). However, the premise for this “beneficial effect” is that ferroptosis is sufficient to cross the threshold for clearing lesion cells. If lesion cells are only in a state of sublethal ferroptosis stress, they may not be truly cleared and instead promote angiogenesis, inflammation amplification, and fibrotic remodeling by releasing factors such as VEGFA and IL-8 (239, 240). Therefore, regarding lesions, the assumption that “more ferroptosis is better” is inaccurate; the critical factor is whether ferroptosis achieves an effective clearance level without inadvertently promoting remodeling.

On the other hand, when ferroptosis occurs in reproductive-related normal cells such as ovarian granulosa cells, oocytes, and early embryos, its consequences are mainly manifested as damage and impaired function, representing the “harmful side” of ferroptosis. The ovarian environment of EMS patients is chronically exposed to excessive iron load and oxidative stress. Ferroptosis can significantly damage granulosa cells and oocytes, reduce follicular development potential and embryo survival rates, and lead to reproductive dysfunction and infertility (241). For example, iron overload-induced ferroptosis can reduce embryo development potential, and the application of the ferroptosis inhibitor Ferrostatin-1 (Fer-1) can partially reverse this damage (242). In this context, the main determinant of ferroptosis is no longer lesion control but the vulnerability of germ cells to oxidative stress and whether their anti-ferroptosis reserves are sufficient to maintain the homeostasis of the follicle–embryo unit (243, 244). Therefore, the same ferroptosis stimulus may have therapeutic potential in ectopic lesions but may directly translate into reproductive toxicity in granulosa cells and oocytes.

Ferroptosis also affects immune cells, further complicating its bidirectional effects. For example, ferroptosis in CD8^+^ T cells at EMs lesions can lead to a decrease in their number and function, weakening the immune clearance of ectopic endometrial cells and promoting disease progression (245); moderate regulation of macrophage ferroptosis may remodel the local inflammatory microenvironment, but current evidence mainly comes from preclinical studies. Overall, in effector immune cells such as CD8^+^ T cells, ferroptosis tends to be harmful, and its phenotype can be characterized by indicators such as downregulation of GPX4, increased lipid peroxidation, and reduced cell activity and cytokine secretion. Therefore, the true therapeutic logic of ferroptosis in EMS should not be summarized as “systemically inducing ferroptosis” but rather understood as “achieving localized, sufficient, and lethal ferroptosis in lesion cells while minimizing off-target exposure to the ovaries, germ cells, and key immune effector cells.”

**Figure 5:**
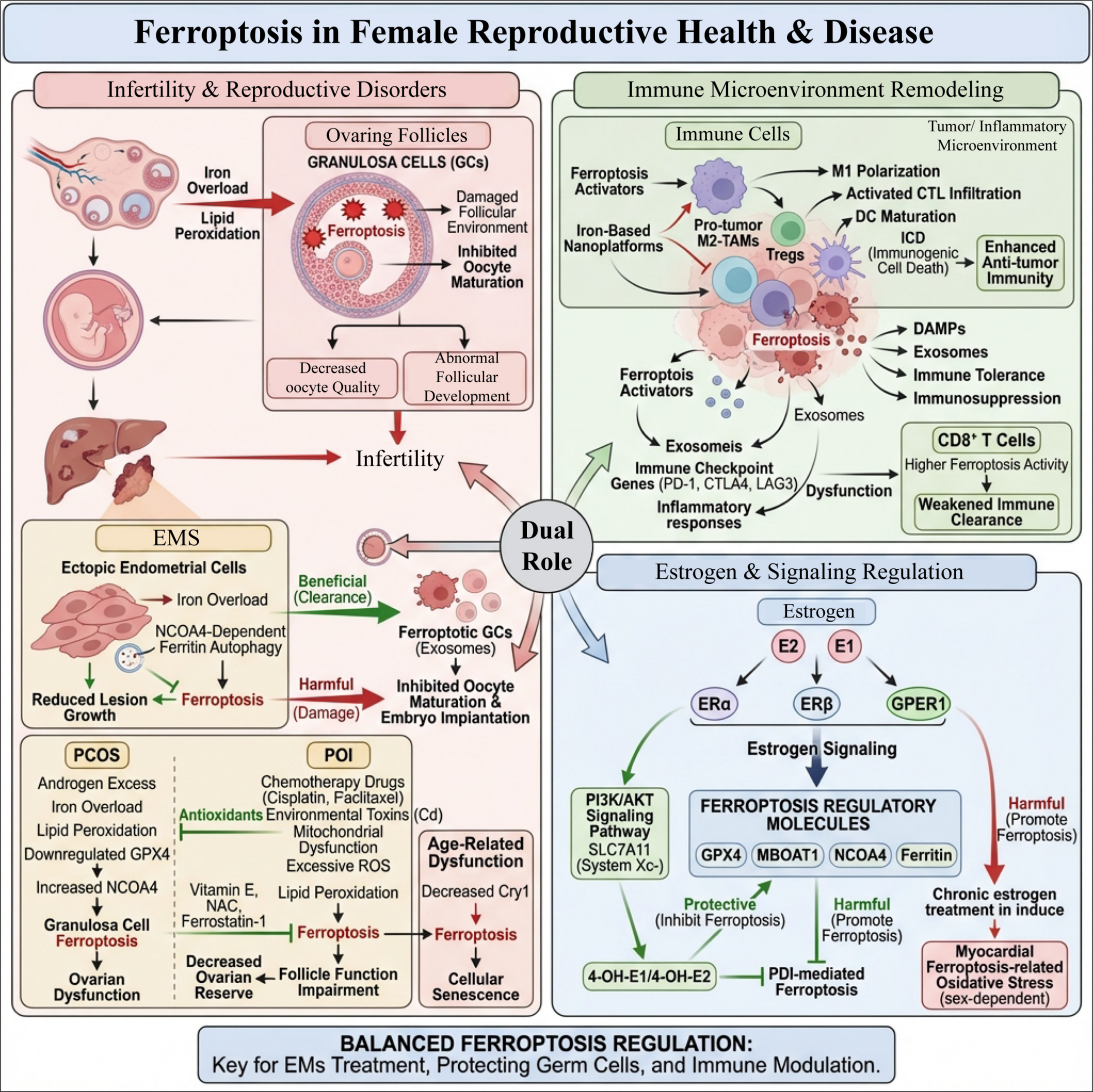
The dual role of ferroptosis in female reproductive health and disease. This image summarizes the multi-layered role of ferroptosis in the female reproductive system. At the reproductive level, iron overload and lipid peroxidation can induce granulosa cell ferroptosis, thereby impairing follicular development, oocyte maturation, and embryo implantation, ultimately contributing to infertility and reproductive disorders; in diseases such as EMS, PCOS, and POI, ferroptosis may both suppress ectopic lesion growth and aggravate ovarian injury and reproductive dysfunction. At the immune level, ferroptosis-related signals can remodel the immune microenvironment by influencing macrophage polarization, T-cell function, and inflammatory versus immunosuppressive states. At the endocrine level, estrogen and its receptor pathways regulate ferroptosis sensitivity through key molecules such as GPX4, SLC7A11, NCOA4, and ferritin. Overall, ferroptosis acts as a double-edged process in female reproductive diseases, and maintaining balanced ferroptosis regulation may be critical for integrating lesion control, reproductive protection, and immune homeostasis. (EMS, Endometriosis; POI, Primary ovarian insufficiency; COS, Polycystic ovary syndrome; GPX4, Glutathione peroxidase 4; ERα, Estrogen receptor alpha; ERβ, Estrogen receptor beta; GPER1, G protein-coupled estrogen receptor; DAMPs, Damage-related molecular patterns). This image was drawn using BioRender software (https://app.biorender.com).

From a translational perspective, this tissue selectivity does not occur naturally and must rely on clear delivery and stratification strategies (246). Theoretically, more feasible directions include: intraperitoneal local administration, intracystic administration, postoperative lesion bed local sustained-release systems, lesion-retaining nanocarriers, and release platforms responsive to high-iron/high-ROS/acidic lesion microenvironments. The common goal is to increase local drug exposure and lethal lipid peroxidation in ectopic lesions while reducing systemic distribution and ovarian exposure. Conversely, in the absence of evidence for lesion-targeted delivery and ovarian protection, any systemic ferroptosis induction strategy may face the risk of “limited lesion clearance and significant reproductive toxicity” (247). For example, activation of the ATF4–xCT pathway in EMS lesions enhances SLC7A11 expression, thereby conferring resistance to ferroptosis and promoting lesion survival; conversely, inhibiting this pathway increases the sensitivity of lesion cells to ferroptosis (248). Additionally, METTL3-mediated m^6^A modification modulates the susceptibility of EMs cells to ferroptosis by regulating the post-transcriptional fate of ferroptosis-related genes (249). These mechanisms provide potential targets for “inducing ferroptosis in lesion cells while protecting or limiting ferroptosis in reproductive and key immune cells.” Additionally, the extensive crosstalk between ferroptosis and processes such as autophagy and inflammatory responses further increases the complexity of its bidirectional effects. Iron overload can exacerbate tissue damage by promoting autophagy-related ferroptosis (250), while the activation of antioxidant pathways such as Nrf2–Keap1 signaling can inhibit ferroptosis and alleviate tissue damage (251). Various natural products such as astragaloside IV, quercetin, and urolithin C have been confirmed to regulate ferroptosis by upregulating GPX4 or inhibiting lipid peroxidation, thereby exerting protective effects under specific circumstances (252) (**Figure 5**).

In summary, the “double-edged sword” nature of ferroptosis in EMS is fundamentally context-dependent. When occurring in ectopic lesion cells and reaching a localized lethal threshold sufficient for cell clearance, it manifests primarily as a therapeutic effect. Conversely, when occurring in granulosa cells, oocytes, embryos, or key immune effector cells, or when persisting in a sublethal manner, it is more likely to drive disease progression or cause reproductive toxicity. Therefore, if ferroptosis is to be used as an intervention strategy for EMS in the future, the premise is not simply “enhancing or inhibiting ferroptosis” but establishing a stratified and precise regulatory framework based on cell type, disease stage, microenvironment characteristics, and local delivery (**Table 1**).

**Table 1:** The bidirectional effects and monitoring indicators of ferroptosis in different cell types.

Cell type	Direction and main consequences of ferroptosis	Key regulatory axis/representative molecules	Recommended measurable indicators	References
Ectopic Stroma/Epithelial Cells	Mainly beneficial: Induces ferroptosis in a local high iron environment, clears abnormally proliferating ectopic endometrial cells, limits lesion expansion; excessive inhibition of ferroptosis favors lesion survival and recurrence.	ATF4-xCT anti-ferroptosis axis; METTL3 mediates m^6^A regulation of ferroptosis-related genes; ferroptosis inducers such as erastin, β-elemene, etc.	GPX4, SLC7A11, ACSL4 proteins/mRNA; BODIPY-C11 fluorescence, MDA, 4-HNE levels; ferritin, free iron, transferrin saturation, etc. in lesion tissue or peritoneal fluid.	(13, 236)
Ovarian Granulosa Cells	Mainly harmful: Iron overload induces ferroptosis, damages granulosa cells, reduces ovarian reserve and steroid synthesis capacity.	Iron overload – lipid peroxidation; Nrf2–Keap1 antioxidant/anti- ferroptosis pathway; Ferroptosis inhibitors such as Fer-1 can protect granulosa cells.	GPX4, SLC7A11, ACSL4; BODIPY-C11, MDA, 4-HNE; ferritin, free iron, and functional indicators such as E_2_, AMH in follicular fluid.	(241, 242)
Oocytes/Early Embryos	Mainly harmful: Ferroptosis reduces embryo survival rate and developmental potential, leading to adverse reproductive outcomes.	Iron overload-ROS- lipid peroxidation axis; Ferroptosis inhibitor Fer-1 protects embryo development.	GPX4, SLC7A11, ACSL4 in oocytes/embryos; BODIPY-C11 signals, MDA, 4-HNE; ferritin, free iron in culture medium or follicular fluid.	(250, 251)
CD8^+^ T Cells	Mainly harmful: CD8^+^ T cells undergo ferroptosis in the lesion microenvironment, leading to exhaustion and functional impairment, weakening immune clearance of ectopic endometrium.	Iron overload and lipid peroxidation; insufficient expression of antioxidant/anti-ferroptosis molecules (GPX4, SLC7A11, etc.).	GPX4, SLC7A11, ACSL4 in peripheral blood or lesion-infiltrating CD8^+^ T cells; BODIPY-C11, MDA; ferritin, free iron, and cytotoxic molecules such as Granzyme B, IFN-γ.	(245)
Macrophages	Bidirectional effects: Moderate ferroptosis can clear overactivated pro-inflammatory macrophages and alleviate inflammation; excessive ferroptosis disrupts phagocytosis and repair functions, exacerbating tissue damage.	Iron load- autophagy-related ferroptosis; Nrf2-Keap1 antioxidant axis; interactions between inflammatory factors (TNF-α, IL-6, etc.) and ferroptosis.	GPX4, SLC7A11, ACSL4 in macrophages; BODIPY-C11, MDA, 4-HNE; serum/peritoneal fluid ferritin, transferrin saturation, free iron, and inflammatory factor levels.	(251)

## 6 Microbiome - iron metabolism - cross network of ferroptosis

### 6.1 Gut microbes and systemic iron homeostasis

Gut microbes play an important role in regulating the host’s systemic iron homeostasis, mainly through mechanisms including the regulation of iron absorption and bioavailability by iron carriers and organic acids produced by microbes, as well as the impact of intestinal inflammation on systemic iron distribution (253, 254). Iron is an essential trace element for biological functions, serving as a key factor in host cell metabolism and an indispensable element for the survival and reproduction of gut microbes. Bacteria in the gut efficiently compete for iron resources by secreting siderophores and other iron carriers that chelate free iron. This enhances microbial iron uptake and also affects the host’s intestinal iron absorption efficiency (255). In addition to iron carriers, certain organic acids produced by gut microbes can promote the dissolution and redox conversion of iron by altering the pH of the intestinal lumen, thereby increasing the bioavailability of iron and indirectly promoting its absorption and storage in the host (256, 257). The composition and metabolic activity of gut microbes are closely related to host iron metabolism. The diversity of microbial communities and their metabolic products, such as SCFAs and secondary bile acids, can influence the function of intestinal epithelial cells and regulate the expression of iron transport proteins, thereby affecting transmembrane transport and systemic distribution of iron (253, 258). Furthermore, exosomes produced by gut microbes, carrying iron-regulating molecules, may directly participate in the regulation of host cell iron metabolism, indicating a complex signaling and material exchange between microbes and the host (259). The state of intestinal inflammation is an important factor that disrupts systemic iron homeostasis. The inflammatory process activates immune responses, leading to abnormal elevation of iron-regulating hormones such as hepcidin, which inhibits intestinal iron absorption and the release of iron from cells, resulting in an imbalance in local iron distribution (260). Both iron overload and iron deficiency can trigger dysbiosis of the gut microbiome, increasing the abundance of harmful bacteria such as Mucispirillum, inducing or exacerbating intestinal inflammation, and forming a vicious cycle (261). Additionally, iron overload exacerbates damage to intestinal epithelial cells and inflammatory responses by activating the ferroptosis pathway, worsening intestinal barrier dysfunction (262). Inhibiting intestinal inflammation and regulating the gut microbiome can restore iron metabolism balance and have potential therapeutic value. In summary, gut microbes regulate the absorption and bioavailability of iron by producing iron carriers and organic acids, while intestinal inflammation alters systemic iron distribution by affecting iron-regulating hormones and the ferroptosis pathway.

### 6.2 Reproductive tract microorganisms, local inflammation, and iron deposition

**Figure 6:**
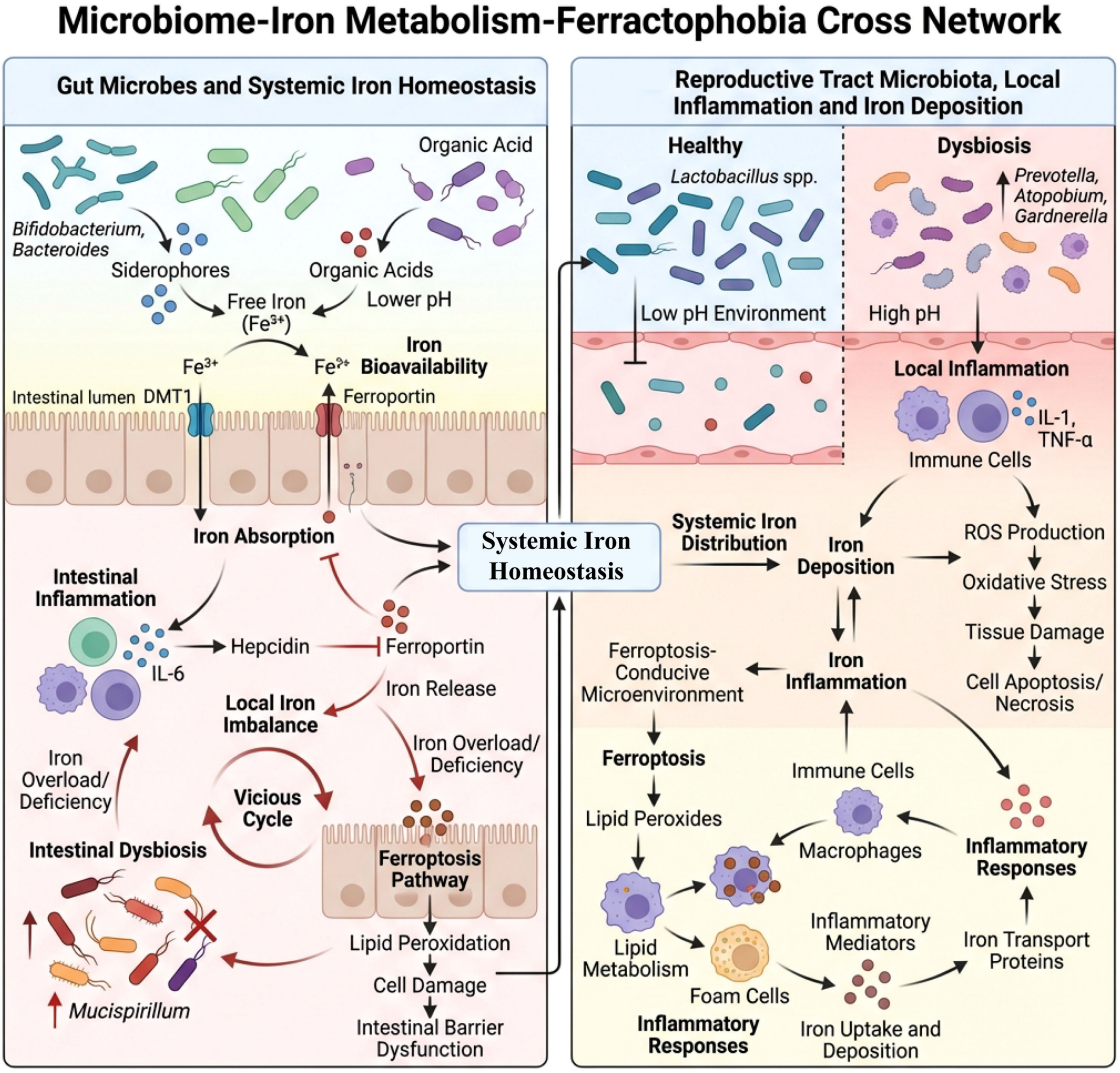
The cross-network of microbiota-iron metabolism-iron death susceptibility. The left side illustrates that the gut microbiota modulates intestinal iron absorption and bioavailability by secreting siderophores, organic acids, and regulating DMT1/ferroportin. Intestinal inflammation and dysbacteriosis upregulate hepcidin, causing local iron imbalance, lipid peroxidation, and ferroptosis, thereby damaging the intestinal barrier and forming a vicious cycle. The right side shows the transition of the genital tract from a healthy state characterized by Lactobacillus dominance and low pH to a dysbiotic microbiota represented by Prevotella, Atopobium, and Gardnerella, accompanied by local inflammation and iron deposition, creating a microenvironment conducive to iron death. Systemic and focal iron imbalance jointly drive lipid peroxidation, macrophage, and immune cell inflammatory responses, exacerbating tissue damage and cell apoptosis/necroptosis. This image was drawn using BioRender software (https://app.biorender.com).

The imbalance of the reproductive tract microbiota (dysbiosis) plays an important role in the pathological mechanism of EMS, especially by promoting local chronic inflammatory responses and iron deposition, creating a microenvironment that facilitates ferroptosis. Under normal circumstances, the reproductive tract microbiota maintains a dynamic balance, primarily dominated by *Lactobacillus* spp., which maintains an acidic environment, inhibits the proliferation of pathogenic bacteria, and protects the mucosa from infection and inflammation (263). Studies have shown that when this microbial balance is disrupted, the diversity of non-lactobacilli increases, and the abundance of bacteria such as *Prevotella*, *Atopobium*, and *Gardnerella rises*, leading to an increase in local environmental pH, triggering inflammatory states and abnormal immune responses (264). Specifically, the local inflammation induced by dysbiosis exacerbates iron deposition through various mechanisms. Iron, as an important trace element, is essential for cellular metabolism. However, its excessive accumulation can catalyze the production of large amounts of ROS, leading to oxidative stress and tissue damage (265). For example, iron deposition has been confirmed to be closely related to persistent local inflammation in chronic venous insufficiency models, where iron drives chronic inflammation and tissue lesions by promoting ROS generation (266). By analogy, similar mechanisms may also exist in EMS lesions. Oxidative stress caused by iron deposition promotes local apoptosis and necrosis of cells. This further activates immune cells and releases pro-inflammatory cytokines, creating a vicious cycle. In addition, iron accumulation and chronic inflammation mutually reinforce each other through mechanisms such as ROS generation and cytokine release, forming a microenvironment conducive to ferroptosis. Ferroptosis is a form of iron-dependent programmed cell death caused by iron overload leading to the accumulation of lipid peroxides that trigger cell death (267, 268). Research has found that in various inflammation-related diseases, local deposition of iron can activate pro-oxidative pathways, inducing cellular ferroptosis and thereby exacerbating pathological damage (269). In EMs, the imbalanced reproductive tract microbiota stimulates the host immune system to produce inflammatory factors, such as IL-1 and TNF-α, promoting excessive accumulation of iron and abnormal expression of iron regulatory proteins, further disrupting iron homeostasis and exacerbating oxidative stress and cellular damage (270, 271). Immune cells, especially macrophages, play a key role in this process. The accumulation of iron in macrophages not only promotes inflammatory responses but also exacerbates local pathological conditions by affecting lipid metabolism and promoting foam cell formation (272). Meanwhile, the release of inflammatory mediators can induce local vascular endothelial cells and tissue cells to express increased levels of iron transport proteins. This facilitates iron uptake and deposition, thereby creating an iron-loaded pro-inflammatory environment (273) (**Figure 6**). In summary, an imbalance in the reproductive tract microbiota induces local chronic inflammation, which disrupts iron metabolism, leads to local iron deposition, and creates a microenvironment that promotes ferroptosis. This microenvironment not only exacerbates tissue damage and lesion development in EMS but also provides potential therapeutic targets for regulating microbiota and intervening in iron metabolism.

### 6.3 Microbial metabolites affect ferroptosis-related pathways

SCFAs, BAs, and indole metabolites, as important metabolic products of gut and reproductive tract microbiota, have been confirmed to play a key role in regulating host cell ferroptosis. Ferroptosis is an iron-dependent form of programmed cell death, whose core mechanism involves lipid peroxidation and the dysregulation of antioxidant defense systems, with NRF2 and GPX4 being the core molecules regulating this process (274). SCFAs, such as acetate, propionate, and butyrate, can enhance cellular antioxidant capacity by activating the AMPK and NRF2 signaling pathways, thereby increasing GPX4 expression, reducing lipid peroxidation, and slowing the occurrence of ferroptosis (275). For example, acetate produced by Akkermansia muciniphila significantly alleviates ferroptosis in metabolic associated fatty liver disease by activating the AMPK/SIRT1/PGC-1α axis in the liver (276). Additionally, SCFAs can improve the intestinal environment and promote the growth of beneficial microbiota, which helps maintain iron homeostasis and reduces the risk of ferroptosis caused by iron overload. Bile acids are important products of microbial metabolism; among them, the secondary bile acid deoxycholic acid (DCA) has been found to promote ferroptosis in high-fat diet-induced intestinal inflammation models. DCA induces iron ion accumulation and lipid peroxidation in intestinal epithelial cells by upregulating HIF-2α and DMT1 expression; this triggers ferroptosis and exacerbates inflammatory responses (277). In contrast, certain microbial metabolites such as oleic acid can inhibit ferroptosis by activating the KEAP1/NRF2/ARE pathway, protecting the liver from ischemia-reperfusion injury. This indicates that microbial metabolites, including bile acid derivatives, have a bidirectional regulatory effect on ferroptosis (278). Indole metabolites derived from tryptophan metabolism, such as trans-3-indole propionic acid and indole-3-lactic acid (ILA), regulate the expression of downstream antioxidant genes such as ALDH1A3 and NRF2 by acting as endogenous ligands for the aryl AhR. They also promote FSP1-mediated synthesis of reduced coenzyme Q10, thereby inhibiting ferroptosis (279). ILA inhibits ferroptosis by activating the AhR/Nrf2 signaling pathway in doxorubicin-induced myocardial injury, demonstrating significant cardioprotective effects (75). These findings reveal that indole metabolites not only play a local immune regulatory role in the gut, but also regulate ferroptosis through systemic signaling pathways, thereby affecting disease progression. Overall, microbial metabolites from the gut and reproductive tract influence iron ion homeostasis and lipid peroxidation levels by regulating the expression of key molecules, such as NRF2, GPX4, and FSP1. This regulation modulates ferroptosis sensitivity and is reflected in various diseases, including metabolic-associated fatty liver disease, colorectal cancer, inflammatory bowel disease, and EMS.

### 6.4 Multi-omics data-supported potential “gut–ferroptosis” axis

In recent years, the development of multi-omics technologies has provided powerful tools for revealing the pathogenesis of complex diseases. EMS, as a chronic inflammatory disease, involves multiple factors such as immune dysregulation, oxidative stress, and cell death, among which ferroptosis, a novel iron-dependent form of programmed cell death closely related to lipid peroxidation, has received widespread attention in EMS and other inflammation-related diseases (280). Multi-omics studies have integrated gut microbiome, metabolomics, and abnormalities in iron metabolism pathways. These studies reveal the complex interactions between gut microbiota and ferroptosis and construct a new perspective on the “gut-ferroptosis” axis (281). Firstly, the gut microbiome, as an important component of host metabolism and immune regulation, influences host iron metabolism and ferroptosis through its metabolites (282). Recent studies have shown that immunosuppressive metabolites produced by gut microbiota can regulate ferroptosis of immune cells through immune cells’ receptors such as the AhR, thereby affecting the occurrence and development of diseases (283). In addition, gut microbiota not only participate in the supply of nutrients, but also influence key metabolic pathways of ferroptosis by regulating lipid metabolism and iron homeostasis (284). For example, certain microbial metabolites such as SCFAs can inhibit ferroptosis through antioxidant and immune regulatory effects, thereby maintaining gut homeostasis (285). Integrated analysis of multi-omics data reveals that under iron overload conditions, the structure of the gut microbiota undergoes significant changes. This leads to an increased abundance of pro-inflammatory bacteria such as Mucispirillum and induces ferroptosis in intestinal epithelial cells, resulting in impaired gut barrier function and exacerbated inflammation. Metabolomics studies focusing on iron overload further found that the metabolite profile of the gut changes, with a decrease in anti-inflammatory metabolites such as α-tocopherol, while the activity of lipid metabolism pathways increases, exacerbating lipid peroxidation and ferroptosis (281). These findings indicate that gut microbiota and their metabolites play a key role in regulating ferroptosis, forming a synergistic network of gut microbiota, metabolites, and ferroptosis that contributes to disease progression. In disease models such as ulcerative colitis, mouse intestinal ischemia-reperfusion injury, and endotoxin-induced intestinal inflammation, regulating the gut microbiota can effectively inhibit ferroptosis, thereby alleviating inflammation and tissue damage (286). Furthermore, specific plant compounds such as hesperidin, rutin, and flavonoids derived from cedarwood can activate the Nrf2/GPX4 signaling pathway to inhibit ferroptosis by regulating gut microbiota and their metabolites, thereby exerting therapeutic effects (287, 288). This suggests that the gut microbiota-ferroptosis axis, as revealed by multi-omics approaches, not only provides a new perspective on the pathological mechanisms of diseases but also offers a theoretical basis for precise interventions targeting conditions like EMS. In summary, multi-omics research integrates abnormalities in microbiomes, metabolic profiles, and iron metabolism pathways. It systematically reveals the mechanisms by which gut microbiota regulate ferroptosis through metabolites, thereby constructing a conceptual “gut–ferroptosis” axis. This axis elucidates the key role of gut microbiota in immune regulation and cell death and provides new insights for studying pathological mechanisms and developing therapeutic strategies for chronic inflammatory diseases such as EMS.

### 6.5 Concept integration: from recurrent bleeding to the “dysbiosis–iron overload–ferroptosis” closed loop

EMS is a chronic inflammatory disease characterized by ectopic endometrial-like tissue, with a complex pathological progression involving multi-layered interactions. During the pathological process, recurrent bleeding is a core pathological feature of EMS, as periodic bleeding from ectopic lesions leads to significant local iron accumulation, forming an iron overload microenvironment (289). This iron overload not only promotes the generation of ROS, further inducing lipid peroxidation and oxidative stress-induced damage, but also triggers iron-dependent programmed cell death-ferroptosis (290). From a microbiota perspective, dysbiosis in the gut and reproductive tract microbiota plays an important role in the pathogenesis of EMS. The gut microbiota regulates host iron metabolism and oxidative stress responses directly or indirectly through its metabolites, such as trimethylamine N-oxide, thereby affecting the state of iron overload and the process of ferroptosis (271). The impaired barrier function caused by microbial imbalance exacerbates local inflammatory responses and immune dysfunction, creating conditions for abnormal iron deposition and forming a vicious cycle in which microbial imbalance, iron overload, and inflammation mutually reinforce each other. The iron overload environment promotes the occurrence of ferroptosis, and ferroptosis itself exhibits a bidirectional effect in the pathological process of EMS. On one hand, ectopic endometrial cells show resistance to ferroptosis by relying on regulatory mechanisms such as the ATF4-xCT pathway, ensuring their survival in a high-iron environment and thus forming lesions and spreading (291). On the other hand, lipid peroxidation and oxidative stress-induced damage caused by ferroptosis lead to functional impairment of surrounding tissues, including ovarian granulosa cells and early embryogenesis, ultimately resulting in decreased fertility (292, 293). Additionally, ferroptosis mediates immune cell dysfunction; for example, ferroptosis of CD8^+^ T cells leads to weakened immune response, promoting the progression of EMS (294). Moreover, the secretion of pro-inflammatory factors and the release of angiogenic factors (such as VEGFA and IL8) induced by ferroptosis further exacerbate inflammation and neovascularization of lesions, worsening the pathological microenvironment (179).

**Figure 7:**
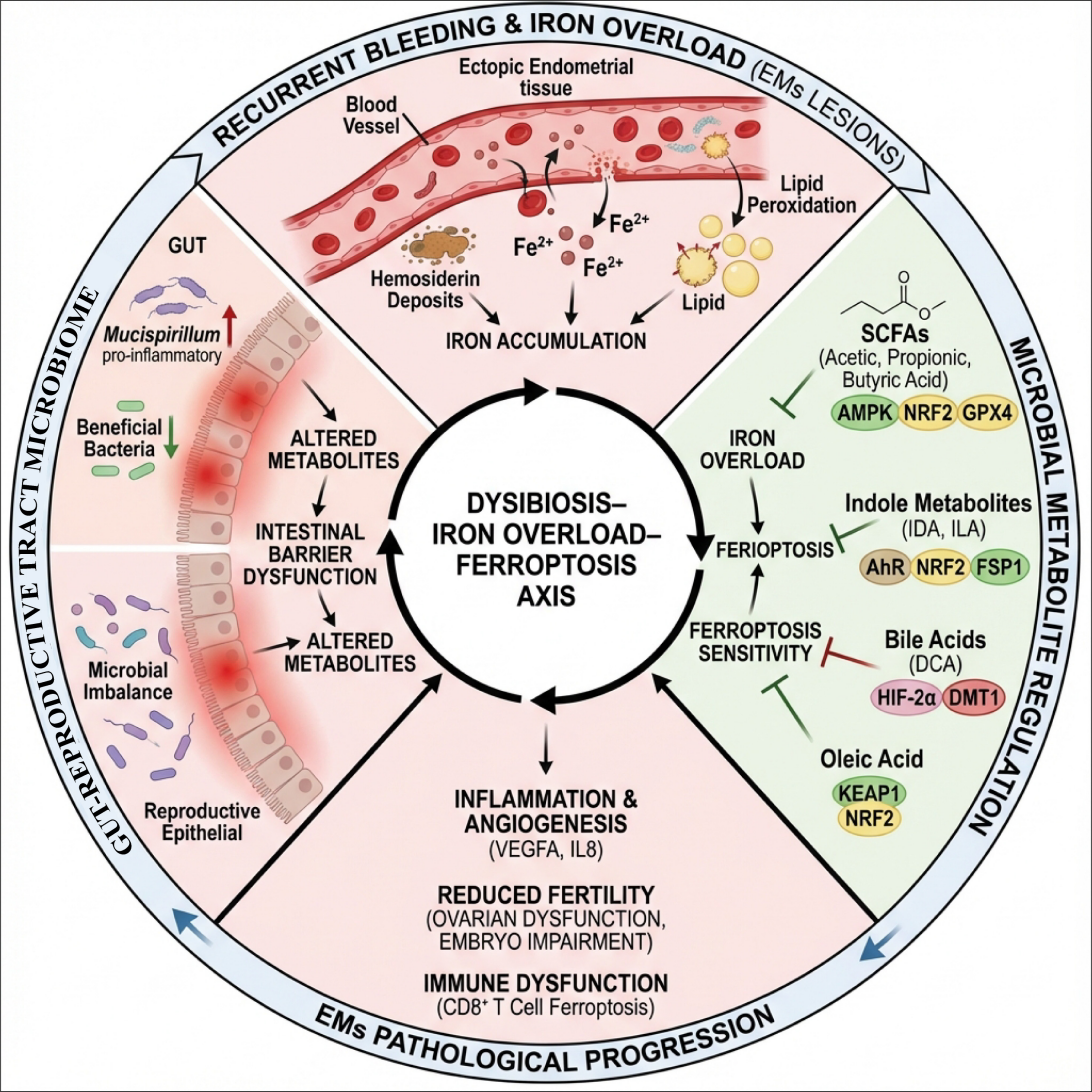
The role of the imbalanced microbiome–iron overload–ferroptosis axis in endometriosis. Recurrent bleeding from ectopic lesions results in the accumulation of hemosiderin and Fe²^+^, triggering lipid peroxidation and enhancing susceptibility to ferroptosis. The imbalance of gut and reproductive tract microbiota together with barrier disruption modulates the metabolite profile, further exacerbating iron overload and ferroptosis. Short-chain fatty acids and certain indole metabolites inhibit ferroptosis through pathways such as AMPK/NRF2/GPX4 and AhR/NRF2/FSP1, while bile acids and fatty acids upregulate HIF-2α/DMT1 or modulate KEAP1/NRF2 signaling to regulate iron homeostasis. These effects manifest as enhanced inflammation and angiogenesis, decreased fertility, and immune dysregulation involving CD8^+^ T cell ferroptosis. (EMS: Endometriosis, SCFAs: Short-chain fatty acids, AhR: Aryl hydrocarbon receptor, VEGFA: Vascular endothelial growth factor A, DMT1: Metal ion transporter 1 antibody). This image was drawn using BioRender software (https://app.biorender.com).

In summary, the pathological process of EMS represents a dynamic network driven by recurrent bleeding, increased local iron load, altered susceptibility to ferroptosis, and dysbiosis of the gut-reproductive tract microbiota. Notably, existing evidence is insufficient to support either of the unidirectional causal relationships that “EMS causes microbial imbalance” or “microbial imbalance precedes and drives the occurrence of EMS.” Instead, a more consistent interpretation with current evidence is that microbial dysbiosis may promote the progression of EMS by disrupting barrier function, modulating immune responses, affecting estrogen metabolism, and disrupting iron homeostasis; conversely, the recurrent bleeding, iron-rich microenvironment, chronic inflammation, and hormonal abnormalities associated with EMS itself may, in turn, shape the gut and reproductive tract microecology. Therefore, the “dysbiosis-iron overload-ferroptosis” framework is more appropriately understood as a bidirectional, mutually amplifying pathological closed loop, rather than a simple linear causal chain. This framework facilitates a more accurate understanding of the persistent progression, propensity for recurrence, and mechanisms of fertility impairment in EMS (**Figure 7**).

## 7 Targeting the microbiome and potential therapeutic strategies for ferroptosis

### 7.1 Probiotics, prebiotics, and dietary intervention

In the pathological mechanism of EMS, the imbalance of gut and reproductive microbiome (dysbiosis) is associated with inflammation amplification, immune deviation, and metabolic disorders. Therefore, microbiome modulation (probiotics/prebiotics/synbiotics and dietary intervention) has a clear theoretical basis and has shown certain potential in preclinical studies. However, it should be emphasized that “direct clinical evidence” in the field of EMS (especially hard endpoints: pain, recurrence rate, fertility outcomes) is still relatively limited. Currently, it should be positioned as a low-risk lifestyle/nutritional adjunct management strategy and a mechanism verification approach, rather than an established “proximal treatment option” or an alternative standard treatment plan.

Probiotics, as active microorganisms, can restore the balance of gut and reproductive microbiome, regulate immune response, and reduce inflammatory response, which some studies suggest may correlate with symptom improvement. However, their definitive efficacy in EMS still needs further verification (295). Research shows that probiotics such as Bifidobacterium and Lactobacillus play a key role in maintaining gut barrier function, regulating the production of SCFAs, and inhibiting pathogenic bacteria growth, suggesting they may help reduce the inflammatory environment associated with EMS (296). Additionally, prebiotics, as “food” for probiotics, can promote the growth and activity of probiotics, further enhancing the stability of the microbiome ecosystem (297). Synbiotics attempt to strengthen effects through the combination of “strains + substrates.” Dietary intervention (such as high-fiber, polyphenol-rich diets) may increase microbial diversity and promote the production of SCFAs, thereby indirectly improving barrier integrity and immune function (298, 299). In contrast, a high-fat/high-sugar Western diet may trigger dysbiosis and low-grade inflammation, exacerbating disease progression. Some preclinical or observational studies also suggest that “dietary pulse foods” like legumes may be related to immune/metabolic improvement (300). However, the transferability of the above evidence has obvious limitations: different studies vary significantly in strains, doses, intervention duration, concomitant treatments, and baseline characteristics of the enrolled population. The causal chain of “microbiome changes → clinical outcome improvement” has not been sufficiently and reproducibly verified. Therefore, a more cautious statement should be that such interventions “may help” improve EMS-related inflammation/metabolic background and provide clues for mechanism research, but cannot yet be regarded as disease-modifying treatments supported by conclusive evidence.

In preclinical models, probiotic interventions have been observed to reduce inflammatory markers, improve microbiome diversity, and affect lesion-related immune cell populations. For example, Lactobacillus rhamnosus OF44 reduced inflammatory factor levels and improved microbiome diversity in animal models (301). However, clinical studies are still generally in the early stages or have limited sample sizes, and individual differences are significant. Future studies should prioritize pre-registered, adequately sized randomized controlled trials with clear endpoints (pain/quality of life, inflammatory markers, recurrence rate, fertility outcomes), while simultaneously conducting concurrent assessment of the microbiome and metabolite profiles to define “effective subgroups,” “optimal time windows,” “dose-response relationships,” and safety boundaries. In summary, probiotics/prebiotics/synbiotics and dietary interventions are supported at the theoretical and preclinical levels, but their positioning in EMS should be considered primarily as adjunct management and tools for mechanism verification, with clinical readiness still needing to be clarified through high-quality trials.

### 7.2 Antibiotics and fecal microbiota transplantation

Antibiotics and FMT can strongly alter the microbial ecology in the short term, making them significant tools in mechanistic research and the study of certain diseases. However, in the context of EMS, the clinical accessibility and risk-benefit ratio of both are still unclear, and it is currently more important to emphasize their attributes as “research tools/exploratory strategies” to avoid being interpreted as directly translatable proximal therapies.

Antibiotics may reduce the burden of EMS lesions in animal models by inhibiting or clearing specific microbial communities, suggesting microbial involvement in disease progression (302). However, it must be emphasized that antibiotics have broad-spectrum effects, which may lead to persistent dysbiosis, increased risk of opportunistic infections, and the development of drug resistance, as well as unpredictable impacts on mucosal immune homeostasis (303). Therefore, in the context of EMS, antibiotics should be viewed more as tools for mechanism research or treatment under specific concurrent infection indications, rather than as empirical long-term medications aimed at “correcting dysbiosis” (304). If future strategies explore “narrow-spectrum/targeted pathogens,” clear evidence of etiology, treatment duration, microbial recovery trajectories after discontinuation, and recurrence risks will also need to be established.

FMT, as a method for directly replacing damaged intestinal microbiota, holds promise for reconstructing the microbial balance in EMS patients and improving the immune environment and inflammatory responses by introducing a fresh microbial ecosystem from healthy donors (305, 306). FMT has shown efficacy in certain diseases, but current research on EMS mainly remains at the preclinical and early exploratory stages (307). However, FMT faces higher translational barriers, including insufficient donor screening and standardization, uncertainty regarding the stability of microbiota colonization post-transplant, and the risk of potential pathogen transmission (especially in patients with complex immune/inflammatory backgrounds) (308). Therefore, FMT should currently be described as an exploratory intervention requiring strict ethical and safety oversight, evaluated within the framework of clinical trials, rather than a routinely recommended treatment.

In terms of risk assessment, both antibiotics and FMT may trigger adverse events such as dysbiosis, opportunistic infections/pathogen transmission, and abnormal immune responses (309, 310). Considering that EMS patients often have complex immune and metabolic states and require long-term management, future related research should strengthen follow-up and adverse event monitoring based on strict inclusion and exclusion criteria and stratification strategies. Researchers should also conduct more cautious precision explorations in conjunction with multi-omics and biological indicators. Given the current evidence, the positioning of antibiotics and FMT in EMS should be limited to mechanism probes and experimental strategies, with their safety and efficacy far from reaching the levels required for clinical promotion.

### 7.3 Targeting iron metabolism and ferroptosis drugs

Drugs targeting iron metabolism and ferroptosis (such as iron chelators and ferroptosis inhibitors/regulators) have demonstrated therapeutic potential in various disease models (311). However, in the context of EMS, it is crucial to strictly differentiate between “cross-disease mechanisms and pharmacological evidence (transferable clues)” and “direct clinical evidence for EMS (which is currently very limited or even lacking).” So far, research on ferroptosis regulation for EMS has primarily been conducted at the cellular, animal, and mechanistic levels, with clinical translation still in the early exploratory stage.

Iron chelators inhibit iron-dependent processes by reducing intracellular free iron, thereby blocking or delaying the occurrence of ferroptosis (312). For example, DFO, as a classic iron chelator, can effectively bind excess iron ions, alleviating oxidative stress and lipid peroxidation caused by iron overload, and has been applied in the treatment of various iron overload diseases (313, 314). However, “clinical application experience” does not equate to “suitability for EMS”; using iron chelators in non-iron overload populations may pose risks such as anemia, iron metabolism disorders, gastrointestinal reactions, or other adverse effects, and their indications, doses, treatment duration, and population screening criteria in EMS have yet to be established (314). Therefore, a more cautious statement in EMS is that iron chelators and other iron metabolism-regulating drugs currently only possess certain mechanistic rationality and cross-disease clues, and do not have an evidence basis for being used as routine treatment options.

Ferroptosis inhibitors such as Fer-1 and Liproxstatin-1 can inhibit lipid peroxidation and demonstrate protective effects in various disease models (314). For instance, in metabolic-associated fatty liver disease (MASLD), iron metabolism disorders lead to iron overload and lipid peroxidation, making ferroptosis an important mechanism in disease progression (316). Ferroptosis inhibitors are considered a potential strategy to slow disease progression; for example, the application of ferroptosis inhibitors has also made progress in diseases such as pulmonary fibrosis and neurological disorders, demonstrating their broad therapeutic potential (317). Additionally, in some infectious diseases and malignant tumors, ferroptosis regulation has also been used as an intervention approach (318, 319). These results suggest that targeting ferroptosis has certain broad-spectrum potential in different diseases, but in EMS, it remains at the level of “candidate targets and drug development directions.”

In summary, drugs targeting iron metabolism and ferroptosis in EMS are currently mainly mechanism-driven candidate directions: on one hand, they can serve as experimental tools for elucidating pathological processes, and on the other hand, they provide ideas for future more refined local delivery or short-term interventions. If they enter the clinical stage, their clinical use should occur under strict safety monitoring within well-designed clinical trial frameworks, based on stratification by iron overload/oxidative stress phenotypes, gradually validating their efficacy and applicable population boundaries, rather than being used empirically in routine clinical practice under current insufficient evidence.

### 7.4 Combined strategy: microbiota, ferroptosis, and traditional treatment

For the management of EMS, traditional therapies such as surgical excision, gonadotropin-releasing hormone agonists (GnRH-a), progesterone, and non-steroidal anti-inflammatory drugs (NSAIDs) remain the mainstream clinical approaches (320). Surgery can directly remove ectopic lesions, alleviate symptoms, and improve fertility; GnRH-a and progesterone suppress the effects of estrogen, reducing lesion activity and inflammatory responses; NSAIDs are primarily used to relieve pain and inflammation (321, 322). However, these therapies are limited in efficacy for some patients and are associated with high rates of disease recurrence, highlighting the necessity to explore new combination treatment strategies (323). In recent years, research has found that imbalances in the gut and reproductive tract microbiota and ferroptosis play key roles in the development and progression of EMS, providing new perspectives for combination therapy. First, gut microbes regulate host immunity and inflammatory responses through metabolites such as butyrate, which can enhance the sensitivity of EMS cells to ferroptosis (8). Studies have shown that the abundance of butyrate-producing bacteria in the gut of EMS patients is reduced, and supplementation with butyrate significantly inhibits the growth of ectopic lesions. The mechanism involves impaired mitophagy mediated by the FFAR2/PPAR-γ/PINK1/Parkin signaling pathway, thereby promoting ferroptosis (324). On the other hand, lipid peroxidation and ferroptosis triggered by iron overload not only damage ovarian granulosa cells, affecting follicular development, but also impair endometrial receptivity, leading to infertility. The role of ferroptosis in the pathological mechanisms of EMS provides potential targets for adjunctive modulation alongside traditional therapies (17, 325). These findings provide a theoretical basis for combination strategies that involve integrating microecological regulation or ferroptosis modulation into traditional treatments.

Based on this, the combined strategy of microbial regulation and ferroptosis modulation with traditional treatment has certain rationality and exploratory space at the mechanistic level, but its clinical feasibility and effectiveness still need to be fully validated (326). For microbiota-dominant EMS, using probiotics, butyrate supplements, or traditional Chinese medicine formulas to restore gut microbiota and improve metabolic disorders and immune environment, while combining with surgery or hormone therapy, may bring additional benefits in some populations, but randomized controlled trials are still needed for evaluation (327). For patients dominated by iron overload, combining iron chelators and antioxidants to reduce iron-mediated oxidative damage should be done with full attention to the adverse reactions and safety monitoring related to iron chelation, and empirical use should be avoided in the absence of sufficient evidence (328). In addition, individualized treatment plans should be based on the pathological mechanism typing of patients, combined with multi-omics indicators such as gut microbiota profile, iron metabolism status, and gene phenotype, to achieve precise combination medication. For example, those with significant dysbiosis and low butyrate levels should prioritize microbiota intervention, while those with high iron load and significant oxidative stress should focus on regulating ferroptosis-related pathways, with traditional therapies adjusted flexibly based on lesion size and fertility needs (329).

In the future, for individualized treatment plans to be truly implemented, it may be necessary to combine multi-omics indicators such as gut microbiota profile, iron metabolism status, and gene phenotype to finely stratify patients, achieving more accurate combination medication design; at the same time, technologies such as nanoparticle drug delivery systems and gene editing are expected to improve targeting while reducing systemic toxicity (330). In summary, the combined application of microbial regulation and ferroptosis mechanisms, along with traditional therapies such as surgery, hormones, and anti-inflammatory treatments, provides a verifiable framework for stratified combined interventions for EMS; however, it should be clarified that its clinical readiness is still limited at this stage, and it needs to be gradually advanced under strict safety assessments and high-quality clinical trial support, avoiding being interpreted as an established “new paradigm of precision treatment” (**Table 2**).

**Table 2:** Emerging therapeutic strategies targeting the microbiome and ferroptosis in EMS.

Intervention strategy	Mechanisms of action	Key evidence/models	Major outcomes	Risks/limitations	References
Probiotics/Prebiotics/Synbiotics & Dietary Interventions	Restore microbiota balance; increase SCFAs; enhance barrier; modulate immunity.	Bifidobacterium/Lactobacillus; high-fiber/polyphenol diets; OF44 improves inflammation & diversity.	Reduced lesion growth; immune modulation.	Limited trials; variability; unclear optimal dose/strain.	(297–300)
Antibiotics	Suppress microbial taxa; short-term dysbiosis correction	Microbiota depletion suppresses lesions in models.	Short-term anti-inflammatory effects.	Broad-spectrum harm; resistance; no optimized protocol.	(302, 303)
FMT	Rebuild microbiota; restore diversity & metabolites; immune improvement.	Animal models: restores diversity, reduces lesions.	Improved inflammation & lesion control (preclinical).	Donor risks; stability issues; limited clinical data.	(307, 308)
Ferroptosis-/Iron-targeting drugs	Iron chelation; inhibit lipid peroxidation; block ferroptosis	DFO reduces ROS/LPO; Fer-1/Liproxstatin-1 effective in multiple diseases.	Protects cells; delays progression	Limited EMS evidence; long-term safety unclear.	(313, 315–317)
Microbial–Iron interactions	Disrupt bacterial iron uptake; ferroptosis protection.	Shown in P. aeruginosa models.	Potential anti-resistance mechanism	Needs disease-specific validation.	(314, 316, 317)
Combined strategies	Butyrate→FFAR2/PPAR-γ/PINK1/Parkin; iron overload→ferroptosis; synergy with standard therapy.	butyrate-producing bacteria; butyrate suppresses lesions; ferroptosis affects fertility.	Higher efficacy; recurrence; fertility benefit.	Need stratification; complex regimens; preclinical evidence.	(328–330)

### 7.5 Safety and translational challenges

With the deepening research on gut-reproductive tract microbiome and ferroptosis in the pathological mechanisms ofEMS, the application prospects of their regulatory strategies in clinical treatment are gradually emerging. However, the safety evaluation and potential toxic risks of microbiome and ferroptosis regulation-based therapies during pregnancy and embryo development remain urgent issues to be addressed (331).

Given that the primary target population for EMS is women of reproductive age, clinical safety should not be merely regarded as a general translational challenge but should be defined as the core prerequisite for whether this field can enter clinical application. It is particularly important to emphasize that ferroptosis induction strategies essentially function by enhancing lipid peroxidation, amplifying oxidative stress, and disrupting iron homeostasis. These processes may also damage oocytes, early embryos, and the endometrial microenvironment required for embryo implantation. Therefore, for patients intending to conceive, particularly during the preconception period and early pregnancy, systemic ferroptosis induction should be considered contraindicated or, at minimum, avoided based on current evidence. In other words, in the absence of sufficient data on reproductive toxicity, embryo toxicity, and teratogenic risks, any systemic ferroptosis induction should not be considered an acceptable intervention pathway for preconception or pregnancy-related stages.

Firstly, although the application of nanomedicine carriers and related biomaterials in female reproductive health offers advantages such as improved drug stability, targeted delivery, and controlled release, it also brings potential adverse effects on fetal development during pregnancy (332). This is mainly because nanomaterials may affect reproductive system function by activating signaling pathways and even exert toxic effects on embryo development. Therefore, systematically evaluating the safety of microbiome regulation and ferroptosis inducers during pregnancy, particularly their impact on embryonic growth and development, is a crucial prerequisite for ensuring the safety of treatment regimens (333). Furthermore, for ferroptosis inducers, risk assessment should not be limited to general organ toxicity but should also focus on the following aspects: (1) whether they affect ovarian reserve, oocyte maturation, and granulosa cell homeostasis; (2) whether they impair fertilization, embryo development, and implantation processes; (3) whether they cause embryo resorption, developmental delay, or structural malformations during early pregnancy; (4) whether systemic agents can cross the placental barrier and adversely affect the long-term health of offspring. Compared to conventional anti-inflammatory or metabolic regulatory drugs, the potential reproductive toxicity and embryo toxicity of ferroptosis inducers warrant greater theoretical concern.

Secondly, the establishment of preclinical research models and long-term follow-up assessments are key to promoting the translational application of such new therapies. Existing preclinical models need to accurately simulate the complex environment of the human reproductive system and the impact of internal and external factors on drug safety, especially under the unique physiological conditions during pregnancy (334). Additionally, long-term follow-up is indispensable for monitoring the chronic toxicity and reproductive health effects of nanomedicines and ferroptosis regulators, enabling the detection of potential late-onset adverse reactions and ensuring the safety of patients and their offspring. Meanwhile, innovative omics technologies, such as nanotoxicology, can identify biomarkers associated with nanomaterials, assisting in risk assessment and safety monitoring and providing scientific evidence for clinical application (335, 336). From a translational perspective, this means that any ferroptosis-based therapeutic exploration in EMS should follow safety thresholds significantly different from conventional drug development: before entering clinical trials, at least systematic assessments of reproductive toxicity, embryo-fetal developmental toxicity, peri-implantation safety, and long-term offspring outcomes should be completed (337); when entering early clinical studies, strict contraceptive requirements, pregnancy exclusion criteria, and reproductive outcome monitoring should also be incorporated into the study design (338). Based on current evidence, systemic ferroptosis induction is more appropriately regarded as a conceptual strategy with clear reproductive risk warnings, rather than a routine treatment direction directly applicable to reproductive-age EMS patients (339).

During clinical translation, microbiome and ferroptosis-related therapeutic strategies also face challenges in standardization and large-scale production. For example, exosomes, as emerging intercellular signaling carriers, show great potential in the treatment of female reproductive diseases. However, their isolation and purification methods are not yet standardized, and long-term safety data are lacking, limiting their widespread clinical application (340, 341). Additionally, drugs regulating ferroptosis, such as β-elemene, have demonstrated promising efficacy in inhibiting EMS, but their molecular targets are complex, and their impact on downstream signaling pathways such as MAPK and STAT3 may lead to unexpected side effects, which must be controlled through in-depth mechanistic research and rigorous safety evaluations (237). Therefore, if ferroptosis regulation is to be considered for inclusion in the EMS treatment framework in the future, a more acceptable direction should prioritize “local, short-term, lesion-restricted” interventions rather than systemic long-term exposure. Theoretically, intraperitoneal local administration, lesion-retention delivery systems, postoperative local sustained-release materials, or targeted carriers responsive to high-iron/high-ROS (reactive oxygen species) microenvironments may better meet the safety requirements of reproductive-age women compared to systemic administration (342, 343); however, even these strategies still require rigorous reproductive safety validation and cannot be assumed safe simply due to their “local delivery” attribute. In summary, promoting the clinical application of gut-reproductive tract microbiome and ferroptosis regulation-related therapeutic strategies not only requires general toxicity monitoring and translational evaluation but must also clearly state that for reproductive-age women, especially those in the preconception period and early pregnancy, systemic ferroptosis induction should currently be considered contraindicated or at least an intervention not recommended. For this field to achieve clinical translation in the future, the prerequisite is not simply proving its “potential efficacy” but first demonstrating its controllability and acceptability in terms of reproductive safety and embryo developmental safety.

Furthermore, this review and related studies still have several inherent limitations that require caution when interpreting conclusions. First, EMS itself exhibits significant phenotypic heterogeneity, with notable differences among patients in terms of lesion sites (ovarian vs. peritoneal types, etc.), disease stages, hormonal exposure, comorbid metabolic/immune conditions, and treatment history. This clinical and biological heterogeneity may obscure or dilute the true effects of microbiome and ferroptosis signals and limits the ability of current evidence to be extrapolated to specific subgroups (344). Second, existing studies vary greatly in microbiome sampling and analysis methods, including fecal vs. reproductive tract specimens, sampling time points, control over confounding factors such as antibiotics/diet, as well as inconsistencies in sequencing platforms, databases, and bioinformatics analysis pipelines. These variations may limit comparability across different cohorts and increase technical bias in conclusions (345, 346). Finally, most current studies are cross-sectional or small-sample cohort studies, and the relationships between microbiome changes, ferroptosis-related molecular abnormalities, and the occurrence/progression of EMS are more correlative, making it difficult to infer clear temporal sequences and causal directions and to determine whether certain changes are pathogenic drivers or “accompanying phenomena” of the disease state (230). Moving forward, future research should also incorporate “reproductive safety stratification” into the design framework. For example, clear distinctions should be made between patients without fertility needs and those with recent pregnancy plans, between systemic exposure and local delivery strategies, and between short-term postoperative adjuvant interventions and long-term maintenance therapy, with corresponding safety endpoint systems established for each (347, 348). Only when both efficacy signals and reproductive safety evidence are robustly demonstrated can ferroptosis-related strategies truly be considered feasible for clinical translation in EMS.

## 8 Conclusion

As a systemic disease, the pathogenesis of EMS cannot be explained by a single factor but is driven by the interaction of multiple networks, including microbiome imbalance, abnormal immune responses, metabolic disorders, and ferroptosis. Recent studies have shown that the gut and genital tract microbiota play an important role in regulating the host immune-metabolic axis, particularly in iron metabolism and ferroptosis pathways, providing a new perspective for understanding the pathological progression of EMS from the holistic dimension of “microecology–iron metabolism–cell death.” It should be noted that existing evidence is insufficient to definitively determine whether EMS drives microbiome imbalance first or whether microbial imbalance precedes and promotes the occurrence of EMS. Based on current human correlation studies, animal experiments, and mechanistic inferences, a more consistent explanation with existing evidence is that there is a bidirectional interaction between the two: microbial imbalance can promote lesion formation and maintenance by regulating immune responses, estrogen metabolism, barrier function, and abnormalities in iron homeostasis, while EMS-related recurrent bleeding, local inflammation, iron-rich environments, and hormonal abnormalities can, in turn, reshape the gut and genital tract microecology. Furthermore, this “gut–endometriosis axis” is not a unidirectional pathogenic chain but more likely a continuous bidirectional circuit of mutual shaping: in addition to the inflammation and metabolic stress caused by the lesions themselves, EMS-related chronic pain, long-term use of NSAIDs, dietary and sleep disturbances, psychological stress, and the low-estrogen state induced by GnRH-a treatment may all act as additional selective pressures, further altering the composition of the gut and genital tract microbiota. In other words, EMS may not only be “driven by dysbiosis,” but its disease burden and treatment process itself may actively exacerbate or maintain dysbiosis, thereby forming a positive feedback loop between pathology and microecology. In this process, the “abnormal immune response” in EMS does not refer to general immune disorders but is mainly characterized by weakened peritoneal immune surveillance, enhanced local immune tolerance, and coexisting persistent low-grade inflammation. Among these, T helper cell imbalance, particularly the remodeling of the Treg/Th17 axis, may play an important role in disease progression: Treg cell expansion or functional deviation can enhance local immunosuppression, compromising the host’s ability to clear ectopic endometrial tissue, thereby promoting its survival, adhesion, and persistent proliferation outside the uterus; while Th17 cells and their related inflammatory factors can further amplify the inflammatory response, promoting microenvironment remodeling, angiogenesis, and lesion maintenance. At the same time, gut and genital tract microbiome imbalance may also regulate Treg/Th17 balance, estrogen cycling, and iron homeostasis through microbial metabolites, inducing local iron overload and triggering ferroptosis, thereby driving lesion formation and persistent inflammatory responses. Iron overload and ferroptosis play a dual role in EMS: on the one hand, iron overload and its related oxidative stress can promote lesion progression and inflammation spread; on the other hand, excessive or uncontrolled ferroptosis may damage ovarian function and weaken fertility, suggesting that this pathway is both an important pathological mechanism and has potential intervention value. Overall, existing studies have preliminarily outlined the mechanistic framework of “dysbiosis–immune remodeling–iron overload–ferroptosis,” but this framework is more appropriately understood as a dynamic bidirectional network rather than a linear model in which microbial imbalance unidirectionally drives EMS. Related evidence is still mainly based on small-sample studies and animal experiments, and there is significant heterogeneity in specific microbial composition, iron metabolism changes, and ferroptosis marker expression. Future research should rely on larger sample sizes, multicenter studies, and multi-omics integration to further clarify the temporal sequence and causal direction between microbial imbalance and EMS, while simultaneously assessing the feedback effects of disease severity, pain burden, drug exposure, and hormonal treatment status on the microbiome. It is also necessary to clarify the pathogenic role and intervention potential of key immune pathways such as Treg/Th17 in the metabolic–gut axis, thereby advancing the field of EMS research from mechanistic studies toward precise classification and clinical translation.

## Glossary

EMS: Endometriosis
ATF4: Activating Transcription Factor 4
NK cells: Natural killer cells
LPS: Lipopolysaccharide
PAMPs: Pathogen-associated molecular patterns
ROS: reactive oxygen species
TLR: Toll-like receptor
AhR: Aryl hydrocarbon receptor
IDO1: 2,3-dioxygenase 1
SCFAs: Short-chain fatty acids
BAs: bile acids
HDAC: histone deacetylase
Tregs: regulatory T cells
FMT: Fecal microbiota transplantation
TF: tissue factor
CMPs: Circulating microparticles
DIE: deep infiltrating endometriosis
cMP-TF: Circulating microparticles carrying tissue factor
ECM: Extracellular matrix
EMT: Epithelial-mesenchymal transition
FN1: fibronectin
HGSOC: High-grade serous ovarian cancer
DFO: Deferoxamine
MDA: malondialdehyde
GPX4: Glutathione peroxidase 4
GSH: glutathione
AMH: anti-Müllerian hormone
HO-1: Heme oxygenase-1
Bach1: BTB and CNC homology 1
ARA: Arachidonic acid
COX: Cyclooxygenase
LOX: Lipoxygenase
PUFA: polyunsaturated fatty acid
VEGFA: Vascular endothelial growth factor A
PCOS: Polycystic ovary syndrome
GCs: granulosa cells
POI: Primary ovarian insufficiency
NAC: N-acetylcysteine
TAMs: tumor-associated macrophages
DCs: Dendritic cells
ERα: Estrogen receptor alpha
ERβ: Estrogen receptor beta
GPER1: G protein-coupled estrogen receptor
PDI: Protein disulfide isomerase
ILA: Indole-3-lactic acid
Fer-1: Ferrostatin-1
MASLD: Metabolic-associated fatty liver disease
GnRH-a: Gonadotropin-releasing hormone agonists
NSAIDs: Non-steroidal anti-inflammatory drugs
FXR: Farnesoid X receptor. DAMPs, Damage-related molecular patterns
DMT1: Metal ion transporter 1 antibody.
